# Berry-Derived Polyphenols in Cardiovascular Pathologies: Mechanisms of Disease and the Role of Diet and Sex

**DOI:** 10.3390/nu13020387

**Published:** 2021-01-27

**Authors:** Rami S. Najjar, Casey G. Turner, Brett J. Wong, Rafaela G. Feresin

**Affiliations:** 1 Department of Nutrition, Georgia State University, Atlanta, GA 30302, USA; rnajjar1@student.gsu.edu; 2 Department of Kinesiology and Health, Georgia State University, Atlanta, GA 30302, USA; chollowed1@student.gsu.edu (C.G.T.); bwong@gsu.edu (B.J.W.)

**Keywords:** polyphenols, berries, sex hormones, sex differences, cardiovascular disease, inflammation, oxidative stress, apoptosis, remodeling, heart failure

## Abstract

Cardiovascular disease (CVD) prevalence, pathogenesis, and manifestation is differentially influenced by biological sex. Berry polyphenols target several signaling pathways pertinent to CVD development, including inflammation, oxidative stress, and cardiac and vascular remodeling, and there are innate differences in these pathways that also vary by sex. There is limited research systematically investigating sex differences in berry polyphenol effects on these pathways, but there are fundamental findings at this time that suggest a sex-specific effect. This review will detail mechanisms within these pathological pathways, how they differ by sex, and how they may be individually targeted by berry polyphenols in a sex-specific manner. Because of the substantial polyphenolic profile of berries, berry consumption represents a promising interventional tool in the treatment and prevention of CVD in both sexes, but the mechanisms in which they function within each sex may vary.

## 1. Introduction

There are sex differences in the prevalence and pathogenesis of cardiovascular diseases (CVD). These differences are likely related to endogenous estrogen effects, as epidemiological data suggest the menopausal transition, which occurs at approximately 50 years of age, is associated with significant changes in cardiovascular health and CVD diagnoses in females. For example, between ages 20–34 and ages 35–44, 13% and 32% of females are diagnosed with hypertension compared to 26% and 43% of males, respectively; however, 65% of females and 66% of males are diagnosed with hypertension between ages 55–64, and diagnoses in females exceed that of age-matched males in higher age brackets [1]. A similar trend is seen with the prevalence of total CVD, including hypertension, coronary artery disease (CAD), heart failure (HF), and stroke, where there is a 17% prevalence in females and 30% prevalence in males between ages 20–39, but prevalence increases to 52% and 57% from ages 40–59 and further to 78% and 77% from ages 60–79, respectively [1]. Regarding pathogenesis, approximately two-thirds of ischemic CVD cases in males are obstructive in nature, whereas two-thirds of ischemic CVD cases in females are non-obstructive [2]. This illustrates a greater atherosclerotic burden in males, especially in larger vessels, but a greater impact of dysregulated vascular function in females, and possibly a larger role for microvascular dysfunction in particular [3,4,5]. Divergent mechanisms of pathogenesis persist into HF as well, where females are more likely to develop HF with preserved ejection fraction (HFpEF) compared to males, who are more likely to develop HF with reduced ejection fraction (HFrEF) [6]. Collectively, this indicates the presence of sex-specific pathways in CVD pathogenesis, which likely includes a specific role for sex steroids and corresponding receptors, given the association with menopause in females. Further, the sex-specific pathogenesis of CVD indicates a potential for sex-specific mechanisms of intervention.

Inflammation, oxidative stress, and pathological remodeling of the cardiovascular system are consistent across a number of CVD-related pathologies and are major drivers of disease [7,8,9]. Biological sex (e.g., female and male) is a non-modifiable CVD risk factor and can influence these pathways through physiological mechanisms [10]. Whether there are sex-specific effects of lifestyle modifications (i.e., changes in diet) in CVD pathogenesis is not well understood. Dietary approaches that emphasize plants are associated with a reduced risk of CVD events [11,12], and consumption of such diets may reduce systemic inflammation [13], attenuate angina, improve endothelial function, reduce atherosclerotic lesions, and improve cardiac function and morphology in humans with CVD [14,15,16]. A major bioactive component of these diets are polyphenols, which likely play a key role in mediating these clinical effects at the cellular level [17]. Berries are an exceptionally rich source of polyphenols, particularly flavonoids and phenolic acids [18]. The dietary inclusion of berries can improve CVD risk factors, including a reduction of blood pressure and serum inflammatory cytokines [19,20], and is associated with reduced CVD mortality [21,22]. Thus, consumption of berries presents a major therapeutic strategy for CVD.

Polyphenol intake is greater in females compared to males, especially polyphenols from fruits and vegetables [23]. In a longitudinal study, males had lower total dietary polyphenol intake and lower total urinary polyphenols than females [24]. Though it is possible that these behavioral factors can influence sex differences in berry polyphenol-related outcomes, sex-specific outcomes are likely more so related to cellular effects of berry polyphenols in cardiovascular health and disease. Because males and females portray differences in cellular mechanisms of inflammation, oxidative stress, and pathological remodeling, the cellular effects of berry polyphenols may also affect CVD pathology in a sex-specific manner. In this review, we will discuss mechanisms of CVD and how polyphenols may serve to sex-specifically mitigate these effects. While we recognize the potential influence of gender (i.e., sociological or cultural gender roles) on human physiology and CVD risk, we will use terms referring to biological sex (i.e., male and female) throughout this review for brevity. Lastly, we focus, as much as possible, on human-based studies; however, in order to explain potential mechanisms, we do make reference to cell culture and animal studies where mechanistic studies in humans are either lacking or may be difficult to perform in humans.

## 2. Berry-Derived Polyphenols

Polyphenols are subdivided into four major classes: flavonoids, phenolic acids, lignans, and stilbenes. In berries, lignans are relatively absent [25] and stilbenes, particularly trans-resveratrol, constitute a small portion of their total polyphenol content [26]. In contrast, flavonoids and phenolic acids comprise the majority of the polyphenolic profile of berries [18]. Flavonoids are subdivided into six major subclasses: flavanols, flavonols, anthocyanins, flavones, flavanones, and isoflavones [27]. In berries, flavanols, flavonols, and anthocyanins are the dominant subclasses. Flavonols contain ketones with a hydroxyl group at position 3 of C ring, while flavanols lack the ketone of flavonols. Further, anthocyanins contain a positively charged oxygen at position 4 of C ring.

Phenolic acids are the most diverse class of polyphenols in berries and are derived in a number of ways. While phenolic acids can be unbound and free in the fruit, they can be derived from (1) metabolic and microbial degradation of flavonoids and (2) hydrolytic liberation of phenolic acid polymers (e.g., ellagitannins) during digestion [28]. Phenolic acids can be classified into two overarching categories, hydroxycinnamic acids, which contain a saturated tail followed by a carboxylic acid, and hydroxybenzoic acids, which lack tail saturation.

### 2.1. Metabolism of Berry-Derived Polyphenols

Polyphenol parent compounds are rarely detected in serum following consumption [29,30], as they are hydrolyzed into metabolites in the small intestine and undergo significant structural modifications by the gut microbiota and in the liver [31,32]. For example, 29 different metabolites were identified postprandially in serum of male subjects who consumed a 500 mg oral bolus of cyanidin-3-glucoside [30]. Polyphenols in the microbiome undergo a plethora of chemical reactions, including C- and O- glycosylation, hydrolysis of esters, dehydroxylation, and demethylation, all of which increase intestinal absorption [32]. Ellagitannins, rich in strawberries, blackberries, and raspberries [33,34], are hydrolysable polymers of ellagic acid and gallic acid. However, in the presence of gastric enzymes, hydrolysis does not occur to liberate ellagic and gallic acid, and instead microbial metabolism liberates these compounds, yielding further metabolism of ellagic acid into dibenzopyranone urolithins A and B. [35]. Following absorption, polyphenols are further modified in the liver, and undergo monooxygenation by cytochrome P450, and these modified compounds are then further catalyzed by phase II enzymes, UDP-glucuronosyl transferases and sulfotransferases, to increase solubility [36]. Isoforms of these enzymes have differing activity in males and females with no clear cumulative trend [37,38]. Further, intestinal transport of polyphenols differs between sexes, as females were found to intestinally hydrolyze quercetin from rutin (quercetin 3-rutinoside) far more efficiently than males, which led to increased serum concentrations of quercetin compared to males [39]. Once in serum, polyphenol metabolites are often albumin-bound, which can lower serum polyphenol concentrations [40], but albumin also stabilizes these metabolites from oxidative modifications [41] and may allow for slower release. This evidence does not suggest that lower concentrations of polyphenols observed physiologically are not efficacious. For example, bilberry extracts, which contain a number of anthocyanins, including cyanidin, delphinidin, petunidin, peonidin, and malvidin derivatives, reduced ischemia-reperfusion (I/R) injury in isolated male Wistar rat hearts at somewhat physiologically relevant concentrations of 0.1–1 mg/L [42]. This concentration reduced cardiac arrest, ventricular fibrillations, and tachycardia compared to control animals, but higher concentrations of bilberry (5–50 mg/L) resulted in a dose-dependent worsening of cumulative arrhythmias [42].

Polyphenols almost certainly exert their effects intracellularly due to changes in gene and protein expression (discussed below). However, berry-derived polyphenols are typically hydrophilic due to numerous hydroxyl groups. As such, crossing lipid bilayers poses a challenge. It has been observed that as hydroxyl group count decreases, hydrophobicity for membrane vesicles inversely increases [43]. Polyphenol metabolites, which contain far fewer hydroxyl groups, could passively diffuse across lipid bilayers. Indeed, microbial metabolism of polyphenols can yield metabolites that have been completely dehydroxylated [44,45]. Nonetheless, membrane transporters likely facilitate the uptake of more common hydrophilic moieties. In endothelial cells, bilitranslocase, a cell membrane transporter, was responsible for the uptake of the anthocyanin cyanidin-3-glucoside and corresponded with increased cellular antioxidant activity [46]. Further, in isolated male rat hearts that underwent I/R, cyanidin-3-glucoside significantly improved coronary flow, which was completely blunted by bilitranslocase antibody, preventing the absorption of cyanidin-3-glucoside. Bilitranslocase may also shuttle other anthocyanins, quercetin, and other polyphenols [47,48,49]. ATP-binding cassette transporters and solute carrier transporters may also be of relevance in polyphenol membrane transport, but evidence is ambiguous [50]. Males and females appear to express membrane transporters differently, with mixed findings in both sexes, but the clinical significance of these sex differences is unclear [51]. Further complicating matters, cross-species differences between metabolite appearance in serum and phase II enzyme activity between humans, rats, and mice differ substantially [52]. Thus, utilizing animal models as a proxy for humans to study sex-specific polyphenol metabolism may not be appropriate, and more human studies are needed.

### 2.2. Sex-Specific Effects of Polyphenols on the Microbiota

The gut microbiota is associated with cardiovascular function and disease (reviewed extensively by Battson et al. [53] and Witkowsky et al. [54]). Physiologic factors, such as age, biological sex, and hormone status, influence characteristics of the microbiota, such as diversity and abundance [55,56,57,58]. Females have increased microbiota diversity [56], although mixed observations have been observed regarding the abundance of *Bacteriodetes* (tend to be beneficial) versus *Firmicutes* (tend to unfavorable) in both males and females [57,59,60]. Hormone status or age may modulate these differences [55,58], as microbiome parameters are similar between males and postmenopausal females but not similar between postmenopausal and premenopausal females [55]. However, one of the greatest determinants of microbiota diversity and abundance of bacterial taxa is diet. This change in diversity can be observed within mere days. For example, in both males and females, switching from a conventional diet to a fiber-rich plant-based diet increased beneficial genus *Prevotella,* while switching to a low-fiber animal-based diet reduced *Prevotella* within four days [61]. Increased *Prevotella* corresponded with increased short-chain fatty acid synthesis, potent anti-inflammatory compounds which can attenuate CVD pathogenesis [62]. Considering the significant contribution of microbial metabolism in polyphenol metabolite generation, it is likely that berries modulate microbiota diversity in a positive manner.

Indeed, a number of berry polyphenols can positively improve gut microbiota diversity in animal models. For example, in male C57BL/6J mice fed a high-fat diet, supplementation of 200 mg/kg/d of blueberry extract for twelve weeks resulted in the reduction of *Clostridium*, a pathogenic microbe, compared to high-fat diet control animals [63]. Peculiarly, *Prevotella* also significantly decreased in blueberry extract-supplemented animals. Nonetheless, cardiac hypertrophy was significantly reduced as assessed by reduced heart weight. In high-fat diet-fed Wistar rats (sex unspecified), 30 mg/kg/d of quercetin for six weeks significantly decreased the *Firmicutes/Bacteroidetes* ratio, which corresponded with increased intestinal short-chain fatty acid production of acetate, propionate, and butyrate [64]. In male Fischer F344 rats treated with dextran sulfate sodium, an inducer of colitis, 1 mg/kg/d of resveratrol increased intestinal *Lactobacillus* and *Bifidobacterium* microbes compared to control animals [65], and these microbes are associated with reduced CVD risk factors [66,67]. Positive changes to microbiota in animal models due to berry polyphenols reflect positive clinical changes in human studies utilizing polyphenol-rich foods. For example, in overweight and obese males and females, 450 mg of pomegranate extract increased butyrate-producing *Faecalibacterium*, *Butyricicoccus,* and *Butyricimonas*, representing a positive microbial shift [68]. Additionally, the consumption of 494 cocoa flavanols for four weeks in healthy males and females significantly increased beneficial *Lactobacillus* and *Bifidobacterium* populations, while *Clostridium* significantly decreased [69]. In a separate investigation in males with metabolic syndrome, the 30-day consumption of 272 mL/day of red wine that contains a polyphenol profile similar to that of berries (rich in flavanols, flavonols, anthocyanins, and stilbenes), significantly reduced the *Firmicutes/Bacteroidetes* ratio and increased *Faecalibacterium, Lactobacillus,* and *Bifidobacterium* [70]. Lastly, a pilot study indicates a trend towards an increase in microbiota diversity in older females, who were not specified as postmenopausal but ranged from 65–77 years old, following six weeks of a blueberry-enriched diet, but there was no observed difference with the intervention in younger females (21–39 years) [71]. Considering the basal differences in microbiota population between sexes, it is likely that a sex-specific polyphenol effect does exist; however, systematic investigation of sex differences in polyphenol-mediated microbiota alterations are currently limited in humans but warranted.

### 2.3. Berry Polyphenol and Estrogen Receptor Interaction

There are physiological differences between males and females that impact many processes, including CVD pathogenesis, in a sex-specific manner [72]. Although females and males produce estrogens and androgens and possess estrogen and androgen receptors (ER and AR, respectively), there are sex differences in circulating sex steroid concentrations and downstream receptor-mediated signaling pathways. ER and AR are expressed throughout the cardiovascular system, in vascular smooth muscle cells (VSMC), endothelial cells, cardiomyocytes, cardiac fibroblasts, monocytes, macrophages, and other immune cells [73,74,75] in both sexes. Therefore, mediation of ER and AR can contribute to cardiovascular health and disease. 17β-estradiol (E2) is a non-specific ER agonist and the primary estrogen subtype in premenopausal females [76], and E2 is implicated in cardio-protection in premenopausal females. Testosterone is converted to E2 via aromatase, so E2 concentration in males is dependent on both testosterone and aromatase concentrations. E2 elicits several protective effects in cardiac and vascular tissues, primarily through ER-dependent mechanisms. Moreover, acute fluctuations in E2 across the menstrual cycle inversely affect inflammation [77] and oxidative stress [78] and directly affect antioxidant status in females [79]. When pertinent, this review will focus on effects of endogenous E2 and will not discuss synthetic estrogens or progestins, such as in oral contraceptives.

There appears to be nearly equivalent ER concentration across the cardiovascular system of male and female rats [80] and in myocardial samples of middle-aged, human males and postmenopausal females [81]. ER expression between human males and premenopausal females is currently unknown. Estrogen status is implicated in ER expression in humans, with postmenopausal females expressing significantly less ERα than premenopausal females [82]. Further, acute fluctuations in E2 in premenopausal females alter ERα expression, where ERα content was decreased by 30% during the early follicular phase of the menstrual cycle (i.e., low E2) compared to the late follicular phase of the menstrual cycle (i.e., elevated E2) [82]. Serum E2 concentration is under-investigated in human males [83], but one investigation allows a direct comparison of serum E2 in a sample of healthy, human postmenopausal females (*n* = 23), premenopausal females (*n* = 20, 60% of samples were donated during the ‘follicular’ phase), and males (*n* = 18) [84]. Serum parent estradiol averaged 31.2 pM/L (range: 10.3–90.9 pM/L) in postmenopausal females, 287 pM/L (range: 55.7–1507 pM/L) in premenopausal females, and 104 pM/L (range: 58.7–245 pM/L) in males [84]. This could suggest greater ER expression in premenopausal females than males based on estrogen concentration; however, empirical evidence is currently lacking.

Sex-specific polyphenolic action is likely related to ER mediation, possibly due to dynamic competitive binding interactions with E2, though there is evidence of beneficial effects of polyphenols that are ER-independent (discussed in detail below). Non-isoflavone polyphenols, such as ellagic acid, kaempferol, myricetin, epicatechin, and quercetin, may act as phytoestrogens, as they can act as ER ligands. However, it is unclear how ER concentration between sexes may affect polyphenolic actions in humans. Due to the cardio-protective effects of ER, it is possible that polyphenols may be of particular importance in the prevention of CVD in males who tend to be at greater risk. Polyphenol binding to ER occurs due to the structure of the ER binding pocket, in which there is a requirement for a phenolic ring to facilitate binding [85]. The hydroxyl group on the phenol ring of polyphenols further enhances ER binding due to hydrogen bonding within the pocket. This indicates that non-isoflavone polyphenols may induce estrogenic or antiestrogenic effects, as they may induce cellular responses similar to that of endogenous E2 or limit endogenous E2 effects by competing for ER binding sites. Some berry polyphenols possess differing ER subtype affinity. For instance, caffeic acid and quercetin have a greater binding affinity for ERβ than ERα [86,87], yet ellagic acid can bind more strongly to ERα and antagonizes ERβ [88]. Therefore, polyphenolic ER subtype selectivity may also contribute to sex-specific outcomes and yield cellular effects in an ER-dependent manner.

**Figure 1:**
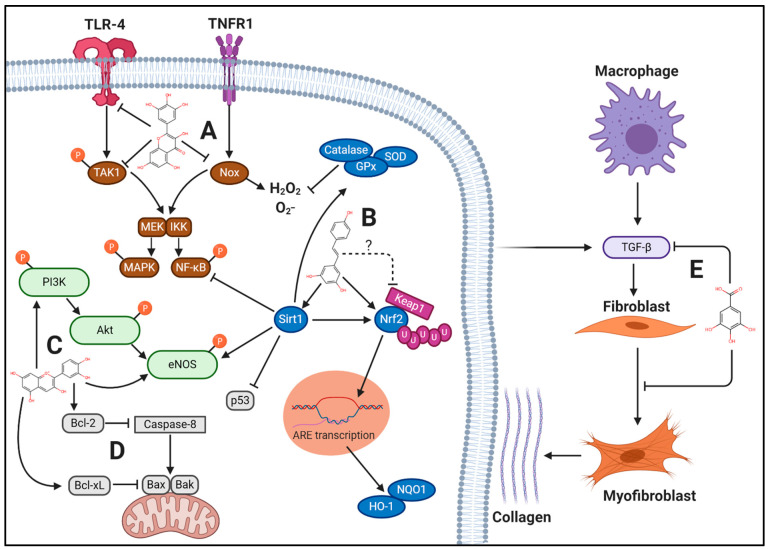
**Mechanisms of Berry Polyphenols in CVD**. Polyphenols found in berries can attenuate the pathological cellular processes of cardiovascular disease (CVD) in a multi-targeted manner. (**A**) Berry polyphenols, such as myricetin, reduce inflammation by decreasing toll-like receptor (TLR)-4 activation and phosphorylation of transforming growth factor-β (TGF-β)-activated kinase 1 (TAK1) while also mitigating tumor necrosis factor receptor 1 (TNFR1)-dependent NADPH-oxidase (Nox) activation. Thus, phosphorylation of mitogen-activated protein kinase kinase (MEK) and IκB kinase (IKK) and redox modifications are reduced, preventing downstream activity of mitogen activated protein kinase (MAPK) and nuclear factor kappa-light-chain-enhancer of activated B (NF-κB), respectively. (**B**) Berry polyphenols, such as resveratrol, reduce oxidative stress by increasing nuclear factor erythroid 2-related factor (Nrf2) protein expression via a number of mechanisms, of which include possible reduction of kelch-like ECH-associated protein 1 (Keap1) binding affinity to Nrf2, reducing Keap1-mediated ubiquitination. Increased Nrf2 nuclear translocation results in increased transcription of heme oxygenase (HO)-1 and NADPH quinone dehydrogenase (NQO1) due to antioxidant responsive element (ARE) transcriptional activity. Additionally, berry polyphenols increase sirtuin 1 (Sirt1) protein expression, which targets a number of proteins, reducing NF-κB and p53 activity while increasing Nrf2 and endothelial nitric oxide synthase (eNOS) activity. Further, Sirt1 increases antioxidant enzymes superoxide dismutase (SOD), glutathione peroxidase (GPx), and catalase, which neutralize superoxide O_2_^−^ and H_2_O_2_. (**C**) In endothelial cells, berry polyphenols, such as cyanidin, increase phosphoinositide 3-kinase (PI3K) activity, resulting in increased phosphorylation of protein kinase B (Akt) and eNOS, resulting in increased nitric oxide (NO), promoting vasodilation. Additionally, berry polyphenols preserve eNOS protein expression under a number of pathological stimuli, including due to TNFR1 activation. (**D**) Berry polyphenols reduce apoptosis by increasing B-cell lymphoma 2 (Bcl-2), reducing caspase-8 mediated activation of Bcl-2-associated X protein (Bax) and Bcl-2 homologous antagonist killer (Bak), while Bcl extra-large (Bcl-xL) reduces Bax/Bak oligomerization. (**E**) Berry polyphenols, such as gallic acid, reduce fibrosis by decreasing TGF-β protein expression and prevent fibroblast differentiation to myofibroblasts, resulting in reduced collagen synthesis. Created in BioRender.com.

**Figure 2:**
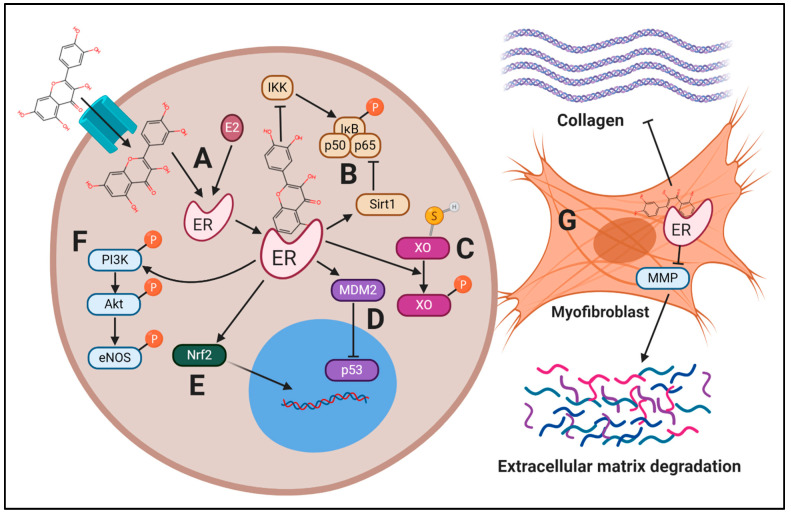
**Potential estrogen receptor-mediated effects by berry polyphenols in CVD.** Multiple cellular targets are mediated by the estrogen receptor (ER), acting in an overall protective manner. (**A**) Polyphenols derived from berries, such as quercetin, can enter the cell via bilitranslocase and act as ER ligands in a similar fashion to 17β-estradiol (E2). (B) Once ER is activated, it inhibits IκB kinase (IKK)-mediated IκB phosphorylation and degradation, preventing nuclear translocation of NF-κB subunits, p65 and p50. Additionally, ER recruits Sirt1 to induce deacetylation of nuclear factor kappa-light-chain-enhancer of activated B (NF-κB), further inhibiting its activity. (**C**) The active sulfhydryl form of xanthine oxidase (XO) undergoes posttranslational modifications by ER, hydrolyzing the sulfhydryl group and inducing phosphorylation of XO, inhibiting its activity and reducing ROS. (**D**) Murine double minute 2 (MDM2) activity is upregulated by ER, leading to increased p53 degradation, reducing apoptosis. (**E**) ER increases nuclear factor erythroid 2-related factor (Nrf2) nuclear activity, increasing endogenous detoxifying enzymes. (**F**) Activity of endothelial nitric oxide synthase (eNOS) is increased due to phosphorylation by phosphoinositide 3-kinase (PI3K)/protein kinase B (Akt) mediated by ER. (**G**) Fibrosis is reduced in myofibroblasts due to ER-mediated effects on matrix metalloproteinase (MMP) and collagen, decreasing the expression of these proteins. Thus, berry polyphenols may be protective partially due to ER-dependent activity. Created in BioRender.com.

In the context of CVD, berry polyphenols target a variety of cellular pathways that drive these disease processes forward (Figure 1), including: (1) reducing inflammatory signaling, (2) reducing oxidative stress, and (3) reducing apoptosis and pathological remodeling. As suggested above, polyphenols may modulate these pathways in a sex-specific manner, facilitating targeted therapies in CVD. The majority of preclinical investigations using berry polyphenols to treat CVD have been performed with male animals, but polyphenols likely exert differing effects between sexes in a multifactorial manner, including ER-mediated effects (Figure 2). Supplementary Table S1 displays the search terms used for study compilation in this narrative review.

## 3. Inflammatory Signaling

In CVD, inflammation is a major driver of atherosclerosis, endothelial dysfunction, hypertension, and HF. In particular, activation of toll-like receptor (TLR)-4 and tumor necrosis factor receptor (TNFR) and their downstream signaling of mitogen activated protein kinase (MAPK) and nuclear factor kappa-light-chain-enhancer of activated B cells (NF-κB) are primarily responsible for these deleterious effects [89,90,91,92,93,94]. NF-κB phosphorylation and nuclear translocation facilitates inflammatory cytokine and chemokine production, promoting leukocyte recruitment and infiltration into the endothelium and myocardium, as well as breakdown of the extracellular matrix and collagen deposition. In conjunction with NF-κB, MAPK phosphorylation facilitates VSMC and cardiac hypertrophy as well as cellular apoptosis [95,96,97,98,99,100,101].

### 3.1. Toll-Like Receptor-4

TLR-4 is found ubiquitously in the cardiovascular system and has a number of ligands. While classically activated by exogenous lipopolysaccharide (LPS), including low concentrations of LPS due to a high-fat meal [102], a variety of endogenous ligands are likely also relevant in CVD, including palmitate [103], oxidized low-density lipoprotein (LDL) [104], fibrinogen [105], and cardiac myosin [106]. Of note in TLR-4 activation is the adaptor protein myeloid differentiation factor (Myd88), which leads to recruitment of TNF receptor-associated factor-6 (TRAF6) and activation of transforming growth factor-β (TGF-β)-activated kinase 1 (TAK1) [107]. TAK1 is responsible for NF-κB and MAPK activation by phosphorylating upstream effectors IκB kinase (IKK) complex and mitogen-activated protein kinase kinase (MEK), respectively. E2 has been shown to inhibit LPS gene products by downregulating NF-κB signaling [75,108]. Sex differences in TLR-4 expression remain unclear, but it appears that E2 and testosterone may affect TLR-4 expression differently based on whether the target tissue is healthy or diseased [75].

While TLR-4 deficient animals are protected from atherosclerosis and HF [109,110], the isolated effects on endothelial function and hypertension are less clear [90,111]. Angiotensin (Ang) II is an oligopeptide and product of the renin-angiotensin-aldosterone system (RAAS), which elicits vasoconstriction classically through Ang II receptor type 1 (AT_1_R). Ang II may interact with glycoprotein MD2 on the TLR-4 receptor independent of AT_1_R, leading to its activation [112]. While TLR-4 blockade reduces blood pressure in spontaneously hypertensive rats (SHR) and deoxycorticosterone acetate-salt sensitive rats, these effects are not always consistent in Ang II-infused animals [90]. It appears that localized TLR-4 deletion in the central nervous system (CNS) is of particular importance in reducing blood pressure by mediating sympathetic and parasympathetic tone, thus reducing cardiac stress [113]. Inhibition of TLR-4 in the CNS results in a reduction of vasoconstricting properties of RAAS, namely reduced systemic norepinephrine, reduced cardiac AT_1_R, and reduced angiotensin converting enzyme (ACE) [113]. However, TLR-4 inhibition in the CNS also upregulated the ACE2 and Mas receptor, which act on the vasodilator side of the RAAS axis [113]. ACE2 catalyzes the conversion of Ang II to Ang (1–7) and Mas is the Ang (1–7) receptor. In humans, there are mixed results on Ang (1–7) concentration and associations with clinical outcomes between sexes [114,115].

### 3.2. Tumor Necrosis Factor Receptor

Tumor necrosis factor (TNF)-α is an inflammatory cytokine, which is elevated in those with CVD [116]. TNF-α can elicit cardiomyocyte apoptosis [117], endothelial dysfunction [118,119], and cardiac dysfunction. Additionally, deletion of the TNF gene from VSMCs improves microvascular function and improves blood pressure [120]. Interestingly, the detrimental effects of TNF-α appear entirely reversible upon removal [121]. While TNF-α is an inducer of NF-κB and MAPK signaling, these effects appear to be primarily mediated via increased NADPH oxidases (Nox)-derived reactive oxygen species (ROS) [122]. TNFR1 and TNFR2 are the major isoforms responsible for TNF-α signaling and have somewhat divergent roles [123]. For example, in a model of ischemic HF, TNFR1 knockout mice were protected from cardiac dysfunction, hypertrophy, and cardiomyocyte apoptosis and had reduced cardiac NF-κB and MAPK protein expression. In contrast, TNFR2 knockout significantly exacerbated these effects due to an attenuation of NF-κB signaling [124]. Increased ROS via TNFR1 appears to be the major difference between these receptors, as TNFR1 can overwhelm protective functions of TNFR2 in cardiomyocytes [123]. The effects of TNF-α and its receptors are likely influenced by sex. In a murine model of cardiomyopathy, transgenic female mice that overexpressed TNF-α had a six-month survival rate of 89% compared to 52% in transgenic male mice [125]. Further, both wild-type males and transgenic male mice had significantly greater TNFR1 expression compared to their female counterparts. These sex differences in TNFR1 may provide some explanation as to why males are at greater risk of CVD compared to females. Cardioprotective effects of TNFR2 are also sex specific. In cardiac I/R injury, TNFR2 in female mice appears cardioprotective by increasing suppressor of cytokine signaling-3, a mediator of pro-inflammatory signaling, whereas in male mice, TNFR2 operates by suppressing the MAPK Jun N-terminal kinase (JNK) [126]. E2 has also been independently associated with suppressing JNK and decreasing downstream TNF gene transcription [127].

In addition to the detrimental effects of inflammation, TNF-α has direct effects on endothelial nitric oxide synthase (eNOS), a nitric oxide (NO) producing enzyme critical to vasodilation and vascular health. TNF-α decreases eNOS mRNA in endothelial cells in a time-dependent manner [128], as well as eNOS protein expression [129], leading to reduced NO metabolite concentration. A negative feedback mechanism exists where NF-κB increases eNOS transcription in response to laminar shear stress, and endothelial NO then inhibits NF-κB, preventing sustained NF-κB activation [130]. E2 positively regulates eNOS and eNOS-derived NO in an ER-dependent manner. In endothelial cells, E2 binding to ER activates PI3K [131,132], leading to protein kinase B (Akt) phosphorylation [132,133]. This results in the phosphorylation of eNOS at Ser1177 [134] and promotes the constitutive production of NO, which is anti-inflammatory at normal physiological concentrations. E2 further prevents activation of NF-κB in endothelial cells by blocking IKK [135]. The role of androgens in anti-inflammatory signaling is less clear. Androgens have been shown to increase TNF-α-induced apoptosis and to induce vascular cell adhesion molecule-1 (VCAM-1) expression, a marker of endothelial inflammation, in endothelial cells through NF-κB signaling, but testosterone has also been shown to phosphorylate eNOS and increase NO production, all in an AR-dependent manner [136]. Normal physiological concentrations of testosterone are generally associated with cardioprotective effects in males [10,137], but females with elevated circulating androgen concentrations are more likely to portray an inflammatory profile [138]. Interestingly, co-incubation of endothelial cells with N-acetylcysteine (30 mmol/L), an antioxidant, for 24 h ameliorated detrimental effects of TNF-α (40 ng/mL) in eNOS protein expression, suggesting a major role of ROS in TNF-α signaling [129]. Additionally, TNF-α signaling inhibits argininosuccinate synthase expression, a critical enzyme in the regeneration of l-arginine, a co-substrate of eNOS [139]. NF-κB signaling also appears to play a role in regulating argininosuccinate synthase expression by decreasing argininosuccinate synthase-DNA promoter binding activity [139].

### 3.3. Berry Polyphenols in Inflammation

Clinically, berries tend to elicit anti-inflammatory effects. For example, Karlsen et al. [140] studied a sample of middle-aged, overweight males (*n* = 47) and females (*n* = 17) with at least one CVD risk factor (hypertension, hyperlipidemia, or smoking). They consumed water or 330 mL of bilberry juice for four weeks, and bilberry juice consumption resulted in a reduction of serum IL-6 and C-reactive protein (CRP), but not TNF-α, all of which are NF-κB transcriptional products [140]. However, in overweight females with metabolic syndrome, the consumption of bilberry extract equivalent to 100 g of fresh berries for approximately four weeks did result in a significant reduction of TNF-α [141]. This suggests possible sex-specific effects, as participants in the trial conducted by Karlsen et al. [140] were mostly male. However, due to the differing disease states within studies, a direct comparison cannot be made. Further complicating matters, due to the unique polyphenol profile of each berry, sex differences may also be apparent depending on which polyphenols are most prevalent. In contrast to Lehtonen et al. [141], which found anti-inflammatory effects with bilberry consumption in females with metabolic syndrome, the consumption of 50 g of freeze-dried strawberry for four weeks did not reduce systemic inflammation, as assessed by high-sensitivity CRP (*hs*-CRP), in a separate investigation of females with metabolic syndrome [142]. However, VCAM-1 did significantly decrease after eight weeks of freeze-dried strawberry consumption in adults (92% female) with metabolic syndrome [143]. Strawberries also did not appear efficacious in obese, hyperlipidemic adults (92% female) in reducing systemic inflammation [144]. Bilberries are a particularly rich source of the anthocyanin petunidin-3-glucoside [145], while strawberries are rich in pelargonidin-3-glucoside [146]; these differing polyphenolic profiles likely account for these anti-inflammatory differences. Nonetheless, in mixed-sex studies that do not include strawberries, berry consumption does appear overall anti-inflammatory. For example, 750 mg of freeze-dried black raspberry or placebo were consumed daily by middle aged males and females for twelve weeks [147]. Following the intervention, serum TNF-α and IL-6 were significantly reduced compared to control [147]. In 72 dyslipidemic participants (75% male, 25% female), consumption of 300 mL of blackberry juice with pulp every day for eight weeks reduced *hs*-CRP [148]. Further illustrating these differences, the consumption of 250 g frozen red raspberries every day for four weeks in diabetic males (20%) and females (80%) significantly reduced IL-6 and TNF-α [149]. Further human studies are needed to elucidate sex differences as it relates to specific polyphenolic profiles found in berries.

Berries likely exert their anti-inflammatory effects by acting as regulators of TLR-4 and TNFR signaling at multiple protein targets. For example, in a pilot study conducted by Nair et al. [150], nine males and ten females with metabolic syndrome consumed 45 g/d of freeze-dried blueberry powder for six weeks. Following the intervention, mRNA isolated from monocytes showed decreased TLR-4, IL-6, and TNF-α gene expression. These effects are mediated by the polyphenols found in berries. For example, in murine RAW 264.7 macrophages, the flavonol quercetin and flavanol catechin were used in isolation and in combination with LPS treatment, a TLR-4 ligand, for 24 h [151]. TLR-4, Myd88, TRAF6, and TAK1 mRNA expression were reduced, and the combination of quercetin and catechin was particularly effective in the reduction of Myd88 protein expression compared to their use in isolation. As expected, phosphorylation of MAPKs, including ERK1/2, p38MAPK, and JNK, were significantly reduced in a similar manner, as was the phosphorylation of p65, the subunit of NF-κB that binds to κB region of DNA encoding inflammatory cytokine transcription. Further, secretion of inflammatory cytokines TNF-α and IL-1β was reduced.

These findings highlight the multiple targets of polyphenols in inflammatory signaling, and the protective effects of polyphenols in macrophages are replicated in models of the cardiovascular system. For example, in human umbilical vein endothelial cells (HUVECs), pre-treatment with 25 μM quercetin for 1 h followed by 50 μM oxidized LDL, a TLR-4 ligand, for 24 h significantly reduced TLR-4 protein expression compared to oxidized LDL treatment alone and also decreased p65 nuclear translocation [152]. Resveratrol also decreased oxidized LDL and LPS-induced inflammation in HUVECs primarily by reducing TLR-4 protein expression [153]. In an ischemic model of anoxia-reoxygenation (A/R) injury, rat cardiomyocytes (donor sex unspecified) were treated with 5, 10, or 20 μM of resveratrol for 5 min following 3 h anoxia and 2 h reoxygenation [154]. TLR-4 protein expression was significantly upregulated due to A/R, but resveratrol decreased TLR-4 in a dose-dependent manner. Further, inhibitor of NF-κB (IκBα), the protein of the NF-κB complex that sequesters p65 into the cytoplasm, was reduced due to A/R, which coincided inversely with increased nuclear p65 translocation. Resveratrol reversed these effects, increasing cytosolic IκBα and decreasing nuclear p65.

This theme is replicated in several in vitro models, as berry polyphenols reduce p65 nuclear translocation induced by Ang II [155] and TNF-α [156] in endothelial cells. In a pressure overload-induced HF model in male mice, the flavonol myricetin (200 mg/kg/d for six weeks) significantly reduced cardiac phosphorylation of TAK1, which coincided with reduced p65 nuclear translocation and reduced phosphorylation of p38MAPK and JNK, but not ERK1/2 [157]. This resulted in significantly improved cardiac ejection fraction and reduced cardiac hypertrophy. Thus, berry polyphenols likely possess potent anti-inflammatory effects, primarily mediated by decreased NF-κB and MAPK activation, with potential sex effects. While not cardiovascular-specific tissue, in breast cancer MCF-7 cells, E2 mitigates NF-κB activity induced by TNF-α signaling via ERα, partially through inhibition of NF-κB acetylation. Resveratrol also partially mediates deacetylation of NF-κB in an ERα/Sirt1-dependent mechanism [158]. As such, the anti-inflammatory activity of polyphenols may be higher in tissues that express ERα [158]. Since ERα is expressed in endothelial cells, VSMCs, and cardiomyocytes, it is likely that resveratrol could disrupt inflammatory signaling in CVD. Because ERα expression is upregulated by higher E2 concentration, the anti-inflammatory effect of resveratrol may be greater in females compared to males, and it is possible that this relationship exists with other polyphenols as well (Figure 2). Further experimental details for some of the investigations cited in this section are displayed in Supplementary Table S2.

## 4. Oxidative Stress

Similar to inflammation, oxidative stress is a characteristic feature of CVD pathogenesis. A balance between ROS production and antioxidant enzyme activity is necessary to maintain normal cellular function. Oxidative stress occurs when the formation of ROS surpasses antioxidant capacity, facilitating NF-κB and MAPK signaling due to upstream redox-sensitive IKK and MEK activation, respectively [159]. Major sources of ROS include Nox and xanthine oxidase (XO). These enzymes produce superoxide (O_2_^−^) and hydrogen peroxide (H_2_O_2_), which contribute significantly to pathological processes in the cardiovascular system, including endothelial dysfunction, hypertension, atherosclerosis, and HF [160,161,162,163,164,165,166,167]. Overall, ROS leads to decreased NO production and bioavailability, increased vasoconstriction, decreased endothelium-dependent and -independent vasodilation, increased inflammation, vascular remodeling, and fibrosis [168]. In general, males have elevated systemic [169], central, and myocardial oxidative stress [170,171] under basal conditions compared to females. This is likely attributable to sex steroids, as castration in female Wistar rats resulted in a 200% increase in myocardial lipid peroxidation [170], demonstrating the protective effects of E2 in oxidative stress.

### 4.1. NADPH Oxidase and Xanthine Oxidase

Oxidative enzymes, such as Nox and XO, are major producers of ROS within the cardiovascular system. Nox1, Nox2, Nox4, and Nox5 are the dominant Nox isoforms in the cardiovascular system, in which all isoforms are present ubiquitously in endothelial cells, VSMCs, and cardiomyocytes [172] at differing concentrations. Nox use oxygen as an electron acceptor, abstracting electrons from NADPH, resulting in O_2_^−^ generation and, in the case of Nox4, H_2_O_2_ generation [173]. Nox is bound to lipid bilayers of organelles or the outer plasma membrane via six transmembrane α-helices. While O_2_^−^ cannot freely pass between lipid bilayers, H_2_O_2_ is able to do so [174]. The micro-environment within the lumen of organelles are under reducing conditions resulting in the spontaneous conversion of O_2_^−^ to H_2_O_2_. Thus, cellular localization of each Nox determines whether O_2_^−^ or H_2_O_2_ will be the predominant end-product. A variety of stimuli can lead to Nox transcription and over-expression, including changes in shear stress [175], Ang II [176], endothelin-1 [177], TNF-α [178], and α-adrenergic receptor agonists [179]. In cerebral arteries of Sprague Dawley rats, reduced Nox activity and lesser Nox1 and Nox4 expression was observed in females compared to males [180]. However, evidence is equivocal in different animal models [180,181,182,183], and human studies assessing sex differences in Nox isoform activity and expression are lacking.

In urea metabolism, nucleotide-derived purines are converted to hypoxanthine (derived from adenosine) and then oxidized by XO to xanthine (derived from guanosine) [184]. XO can then further catalyze the oxidation of xanthine to form uric acid. XO catalysis involves reducing molecular oxygen and the release of ROS in the form of O_2_^−^ or H_2_O_2_. XO is nearly absent from the human heart [185]; however, it is found in abundance in the endothelium [186]. Males with CAD have a 200% increase in arterial XO activity compared with healthy controls [186]. Interestingly, in microvascular endothelial cells, both E2 and E2 stereoisomer (17α-estradiol) reduce XO activity despite the inability of 17α-estradiol to bind to ER, demonstrating ER-independent effects of E2, which may include post-translational modifications of XO [187]. Despite the vasodilator effect of testosterone [188], testosterone also appears to increase O_2_^−^ and peroxynitrite (ONOO^−^) generation and upregulate XO activity through AR-dependent mechanisms [189]. Upregulation of XO can occur due to hypoxia, inflammatory signaling [190], and crosstalk with Nox [191]. Collectively, cardiovascular Nox and XO are implicated in the pathogenesis and/or can be targeted in the treatment of several CVDs [192,193,194,195,196,197], including hypertension, CAD, and HF.

### 4.2. Role of NOS in Oxidative Stress

NOS isoforms can also contribute to oxidative stress. NOS produce NO, which is largely cardioprotective when produced constitutively by the endothelium through eNOS [198,199]. Endothelial NO at physiologic concentrations facilitates vasodilation and decreases vascular resistance by activating VSMC soluble guanylate cyclase (sGC) to produce cyclic guanosine 3’,5’-monophosphate (cGMP) [200]. Endothelial dysfunction is characterized by decreased available NO and is an early indicator of CVD development [201]. Dysfunction of eNOS (i.e., eNOS uncoupling) can contribute to oxidative stress, where eNOS uncoupling produces O_2_^−^ instead of NO [202]. eNOS uncoupling can occur when there is decreased bioavailability of eNOS cofactors, including tetrahydrobiopterin (BH4) and l-arginine. This can occur when there is reduced BH4 synthesis [203] or when BH4 is oxidized or depleted [204]. Further, l-arginine availability can be reduced via diminished argininosuccinate synthase activity [205,206,207,208], increased arginase activity [206,209], or increased asymmetric dimethylarginine (ADMA) production [210]. Argininosuccinate synthase acts in the regeneration of arginine from citrulline, arginase catalyzes the conversion of l-arginine to l-ornithine and urea in the urea cycle [206,209], and ADMA is the result of arginine proteolysis and is a competitive inhibitor of eNOS [211].

The production of NO and eNOS coupling are known to be affected by E2. The binding of E2 to ERα increases stimulatory phosphorylation sites Ser1177 and Ser635 on eNOS, and E2 binding to ERα or ERβ reduces inhibitory phosphorylation site Thr495 on eNOS [212], which aid in the constitutive production of cardioprotective NO. E2 may also prevent eNOS uncoupling and subsequent O_2_^−^ production, potentially by affecting l-arginine and BH4 availability [213,214]. In peri- or postmenopausal females, but not premenopausal females, impaired brachial artery flow-mediated dilation (FMD; a measure of conduit vessel endothelial function) was associated with relative l-arginine deficiency and increased markers of l-arginine metabolism [213], and FMD improved with BH4 supplementation [214], suggesting the reduction in E2 in peri- and postmenopausal women resulted in a decrease in bioavailable precursors required for NO production via eNOS. The effect of androgens on eNOS (un)coupling are still unknown.

When NO is overproduced, such as through activation of inducible NOS (iNOS), it can directly contribute to oxidative stress. iNOS is activated through MAPK and NF-κB signaling under pathological conditions [215,216] and is upregulated in CVDs [217,218,219]. iNOS yields approximately 1000-fold the amount of NO compared to eNOS [217]. Excess NO, which is itself a free radical molecule, is then scavenged by ROS produced from Nox and XO, leading to conversion of NO to ONOO^−^ [218,219]. Nox- and XO-derived ROS and ONOO^−^ oxidize numerous molecules, including sGC. sGC oxidation increases VSMC intracellular Ca^2+^ flux and depletes BH4, which consequently results in endothelial dysfunction [220]. In addition, ONOO^−^ can oxidize low density lipoprotein, which is linked to atherogenic processes, including foam cell formation and leukocyte adherence to endothelial cells [221]. iNOS may represent a pathway of specific interest in females, as E2 non-selectively binds to both ERα and ERβ, but they yield opposing effects on iNOS activation; ERα inhibits while ERβ stimulates iNOS [222]. Testosterone appears to affect iNOS activity differently based on concentration [223]. Macrophages treated with testosterone concentrations of 10^−10^ M and 10^−8^ M showed less iNOS protein compared to those treated with 10^−12^ M testosterone, but iNOS protein treated with 10^−6^ M testosterone was similar to that of 10^−12^ M [223]. This indicates that testosterone concentrations, both low or high, could elicit detrimental effects on iNOS activation and subsequent ONOO^−^ generation.

### 4.3. Endogenous Antioxidant System

Antioxidant enzymes, such as superoxide dismutase (SOD), glutathione peroxidase (GPx), and catalase, also contribute to the redox balance by dissociating ROS. SOD1 (a cytoplasmic enzyme) and SOD2 (a mitochondrial enzyme) catalyze the dismutation of O_2_^−^ into H_2_O_2_ and O_2_ [220]. GPx and catalase both reduce H_2_O_2_ to form H_2_O and O_2_. SOD, GPx, and catalase activity are all inversely associated with CVD risk [224,225,226]. SOD and GPx activity, as well as SOD expression, are also inversely related to atherosclerosis severity [225,226,227]. Both SOD1 and SOD2 upregulation in transgenic mice appear to offer cardioprotective effects in HF, including improved ejection fraction and reduced inflammatory cytokines compared to wild-type mice [228,229]. Further, there is an established relationship between NO and SOD, where SOD protects the bioavailable NO, and NO upregulates the expression of SOD [220,230]. Estrogen affects mitochondrial respiration, mitochondrial ROS production, and antioxidant capacity [231]. Estrogen appears to increase oxidative phosphorylation while simultaneously decreasing mitochondrial production of O_2_^−^ and H_2_O_2_, likely related to estrogenic increases in SOD expression [170,232] and activity [170]. At this time, there is no empirical evidence that physiological doses of estrogen or testosterone influence expression of GPx or catalase [170,232]. Supraphysiological doses of testosterone decrease SOD, GPx, and catalase expression [233], but testosterone deficiency also decreases antioxidant capacity [234].

Other components of the antioxidant system include heme oxygenase (HO)-1 and NQO1. HO-1 catalyzes free heme, a pro-oxidant [235] and major component of a variety of enzymes, to produce carbon monoxide (CO), iron (Fe^2+^), and biliverdin [236]. CO itself has anti-inflammatory effects [237] and acts in a similar manner to NO, increasing cGMP in VSMCs, potentially improving vasodilation [238]. There is greater HO-1 expression and total HO activity in cardiac and aortic tissues of female compared to male rats [239], but sex differences in HO-1 expression or activity in humans is unknown. Additionally, NQO1, an NADPH-reductase, is a ROS scavenger, in part due to the flavin co-factor, which facilitates dismutation of O_2_^−^ to H_2_O_2_ [240]. Further, NQO1 mRNA expression and activity are higher in females compared to males in rats [241], and human data are limited but suggest no sex differences [242]. The principal transcriptional regulators of these antioxidant enzymes are Nrf2 [243] and Sirt1 [240].

#### Nrf2 and Sirt1

Nrf2 is continually synthesized, but it is sequestered in the cytosol by the enzyme Keap1, which undergoes continuous ubiquitination and degradation when the Keap1-Nrf2 complex is formed. Under conditions of oxidative stress, such as excessive H_2_O_2_, reactive cysteine residues on KEAP1 undergo reduction, leading to conformational shifts in Keap1, preventing Nrf2 binding [244]. As such, Nrf2 is able to accumulate in the cytosol and translocate into the nucleus. Similar to NF-κB, Nrf2 binds to anchoring protein CBP, which allows it to bind to the transcriptional region of DNA encoding the antioxidant responsive element (ARE). This leads to the transcription and synthesis of antioxidant enzymes, particularly HO-1 and NQO1 [245]. Because CBP can bind to both NF-κB and Nrf2, these proteins compete for binding [246]. Under conditions of inflammation and oxidative stress, NF-κB appears to bind 10-fold greater to CBP than Nrf2 as observed in HepG2 cells treated with 12-O-tetradecanoylphorbol-13-acetate (PMA; a protein kinase C activator) [247]. Thus, under pathological conditions observed in CVD, it can be assumed that Nrf2 would be deprived of CBP binding, and under these circumstances, insufficient ARE transcription would occur not eliciting an overwhelming enough antioxidant response to counteract ROS. ER-dependent mechanisms appear to positively modulate Nrf2 activity [248]. For example, in cardiomyocytes that underwent hypoxia followed by reoxygenation, a model of I/R injury, the addition of E2 significantly increased Nrf2 nuclear translocation [249]. Conversely, ICI 182,780 (ICI), a non-specific ER antagonist, abolished these effects and reduced Nrf2 protein leading to reduced HO-1 and SOD1 mRNA.

In addition to Nrf2, Sirt1, a redox sensitive deacetylase, has multiple cellular effects, including antioxidant, anti-inflammatory, and anti-apoptotic effects. A direct transcriptional product of Sirt1 activity is SOD2, and Sirt1 increases eNOS, Nrf2, GPx, and catalase expression and inhibits NF-κB activity and apoptotic transcription factor p53 [250]. In animal models of Ang II-induced hypertension and pressure-overload-induced HF, Nrf2 and Sirt1 genetic knockdown exacerbates these conditions [251,252,253,254], while overexpression has protective effects [255,256,257]. As with Nrf2, E2 may also increase Sirt1 protein expression [258], demonstrating the multi-targeted role of E2 on oxidative stress and antioxidant targets. Increasing the activity of these enzymes is of major therapeutic importance in the treatment of CVD.

### 4.4. Berry Polyphenols in Oxidative Stress

Berries appear efficacious in mediating antioxidant status in humans [259]. In a number of acute studies, berry consumption increases plasma antioxidant status [260,261,262,263,264,265,266]. This increase has been shown in healthy middle-aged males after consumption of 240 g of bilberries, lingonberries, or blackcurrants [260] and 100 g freeze-dried blueberry powder with a high-fat meal [261,262]. Increased plasma antioxidant capacity has also been shown in females following ingestion of a blueberry beverage, including 200 g of blueberries, 50 g banana, and 200 mL of apple juice, but this was investigated in relation to exercise-induced muscle injury [266]. A number of mixed-sex samples confirm similar findings following consumption of 400 mL of an antioxidant-rich juice (30% white grape, 25% blackcurrant, 15% elderberry, 10% sour cherry, 10% blackberry, and 10% aronia) [263], 400 mL of elderberry juice [264], or 1 kg of strawberries [265]. Results from another mixed-sex sample indicate that acute consumption of cranberry polyphenols (456 mL cranberry leaf extract beverage or 480 mL of cranberry juice cocktail) may positively alter endogenous antioxidant systems, including increases in GPx and SOD activity [267]. This collectively indicates the efficacy of berry polyphenols in acutely improving antioxidant status in both sexes, but additional investigation of the effect of berry polyphenols in females and on endogenous antioxidant systems is warranted.

Berry polyphenols tend to reduce vascular oxidative stress in a number of chronic studies. For example, in an eight-week trial, 48 postmenopausal females with pre- and stage 1-hypertension were assigned to consume 22 g/d of freeze-dried blueberry powder or placebo [268]. Serum concentrations of NO metabolites increased in subjects consuming the blueberry powder by 68%, which was significantly greater than control [268]. Further, systolic and diastolic blood pressure decreased by 7 and 5 mmHg, respectively, whereas blood pressure did not change in control subjects [268]. In a separate investigation, 44 obese females and four obese males with metabolic syndrome consumed 50 g/d freeze-dried blueberries or placebo for eight weeks [269]. Following the intervention, markers of oxidative stress, including serum oxidized LDL and combined malondialdehyde and hydroxynonenal (indicators of fatty acid oxidation) significantly decreased by 28% and 17%, respectively, which was significantly greater than control [269]. In the previously described investigation by Nair et al. [150] in which males and females consumed 45 g/d of freeze-dried blueberry for six weeks, both serum and isolated monocytes expressed decreased total ROS and serum O_2_^−^ compared to placebo. Further, in pre- and stage 1-hypertensive postmenopausal females, 50 g/d of freeze-dried strawberry for eight weeks significantly increased NO metabolites compared to baseline, although these did not correspond with changes in blood pressure at this dose [270].

Sex differences in the efficacy of berry polyphenols likely exist with regard to reductions in oxidative stress. For example, 26 hyperlipidemic males and females consumed 10 g of freeze-dried strawberry for six weeks [271]. Oxidized LDL decreased to a much greater extent in females compared with males, suggesting greater efficacy of strawberry polyphenols in females. In a 12-week study including 44 subjects with prediabetes (70% female), black raspberry consumption (900 or 1800 mg/day) decreased oxidized LDL in a dose-dependent manner [272]. However, it is likely that these suggestive sex differences are polyphenol specific, although further human investigations are needed to assess these differences.

#### 4.4.1. Berry Polyphenols in Sirt1 and Nrf2 Regulation

Berry polyphenols exert cellular antioxidant effects, not merely due to exogenous ROS scavenging, but also by exerting cytoprotective effects, as polyphenols act as effectors for multiple proteins. For example, under basal conditions, quercetin increases Sirt1 mRNA in a dose-dependent manner in HUVECs [273]. Additionally, in HUVECs, the treatment of 10 μM quercetin for 24 h followed by exposure to 150 μg/mL oxidized LDL for another 24 h resulted in reduced Nox2 and Nox4 expression as well as increased eNOS expression compared to oxidized LDL alone [273]. However, Sirt1 siRNA abolished these protective effects. In vivo, similar findings were observed in a diabetic cardiomyopathy murine model (gender not specified), as resveratrol (25 mg/kg/d for five days) significantly increased Sirt1, reduced cardiac hypertrophy, and improved ejection fraction, but this was abrogated by Sirt1-specific cardiac deletion. Nrf2 activity can also be regulated by polyphenols [274]. This effect was attributed to potential binding to Keap1, causing conformational shifts that prevent Nrf2 from binding to Keap1, thus leading to increased cytosolic concentration of Nrf2 and subsequent nuclear translocation. Computational studies revealed that kaempferol may directly bind to Keap1 in this fashion and prevent Nrf2 binding [275]. However, in both human and rat-specific proteins, it was revealed that gallic and ellagic acid were incapable of binding to Keap1 in the domain associated with Nrf2 binding regulation. Although direct interactions of polyphenols with Keap1 may alter its binding affinity to Nrf2, other interacting proteins that regulate Keap1-Nrf2 complex affinity are also of relevance [276].

For example, p62, a scaffolding protein involved in a variety of cellular processes, such as autophagy, can directly bind to the binding pocket of Keap1 that associates with Nrf2, and thus prevent Nrf2 from binding to Keap1 [277]. In an I/R model of HF in male rats, urolithin B, a downstream microbiome-derived metabolite of ellagic acid metabolism, was injected into the intraperitoneal cavity 0, 24, and 48 h prior to surgery in rats at a concentration of 0.7 mg/kg [278]. Concentrations of p62 in cardiac tissue were significantly decreased in I/R rats that did not receive treatment; however, urolithin B injection significantly abrogated reductions in p62, which was not different than p62 in sham control. This preservation of p62 corresponded with a significant reduction in myocardial infarct size and improved cardiac hemodynamics compared to I/R controls. Further, although nuclear Nrf2 was increased in I/R control compared to sham, urolithin B treatment elicited roughly three-fold greater nuclear Nrf2 compared to I/R control. Increased NQO1 and HO-1 expression was also observed.

#### 4.4.2. Berry Polyphenols in Endothelial Function

Berry polyphenols may also improve endothelial function mediated by reduced oxidative stress. In endothelial cells, exposure to the anthocyanin cyanidin-3-glucoside (5, 10, or 20 μM) for 2 h followed by 1 μM Ang II for 24 h resulted in a significant attenuation of ROS, reduction of iNOS, and increase in nuclear Nrf2 translocation, which coincided with increased HO-1 and SOD in a dose-dependent manner compared to Ang II alone [155]. These positive in vitro effects in endothelial cells reflect in vivo findings. For example, in male mice fed a high-cholesterol, high-fat diet with or without cyanidin-3-O-β-glucoside (2 g/kg) for eight weeks, aortic O_2_^−^ was significantly reduced and cGMP was significantly increased due to anthocyanin supplementation [279]. The anthocyanin-supplemented diet also resulted in improved endothelium-dependent vasodilation and increased phosphorylated eNOS. In a separate investigation, fourteen weeks of supplementation with the phenolic acid ellagic acid (30 mg/kg/d) reduced atherosclerotic lesion area in the aortic arch of high-fat diet-fed male mice compared to mice with no phenolic acid supplementation. Additionally, aortic Nrf2 and HO-1 were significantly increased, and aortic NOS activity was significantly higher with ellagic acid supplementation [280]. Preservation of eNOS due to berry polyphenols under pathological stimulus is a common theme. Cyanidin, for example, increased eNOS protein under basal conditions and also abrogated TNF-α-induced transcriptional downregulation of eNOS in endothelial cells [281]. In male SHR, gallic acid supplementation (1% of drinking water) for sixteen weeks significantly reduced aortic components of RAAS, including ACE and AT_1_R expression, compared to control SHRs [282]. Additionally, blood pressure and left ventricle (LV) Nox2 protein expression were significantly reduced, and this coincided with reduced LV hypertrophy. Gallic acid also appeared protective in Ang-II infused male mice (490 ng/kg/min) [283]. After two weeks of infusion, gallic acid dose-dependently (5 and 20 mg/kg/d) improved blood pressure and increased aortic total eNOS and phosphorylated eNOS protein expression. In excised aortas from male SHR that consumed an 8% blueberry diet for eight weeks, blueberry supplementation dramatically reduced phenylephrine-induced vasoconstriction compared to SHR controls, which was completely abolished by eNOS inhibitor, l-*N*^G^-monomethyl-arginine [284].

A number of berries have clinically demonstrated improved vascular function in humans. In healthy males, the consumption of wild blueberries increased postprandial FMD [285]. Interestingly, a biphasic response was observed, with peak FMD detected at 1–2 h and 6 h, suggesting microbial metabolism of parent flavonoids to yield secondary metabolites at the later time point. Plasma phenolic acids, ferulic acid and caffeic acid, peaked at 1–2 h, while hippuric acid and homovanillic acid peaked at 6 h. These changes in plasma polyphenol concentrations also reflected decreased neutrophil Nox activity, which significantly decreased at 1–2 h and 6 h. Also, in young males, 200 and 400 g of red raspberries significantly improved FMD at both 2 and 24 h compared to control [286]. Ellagic acid serum concentrations peaked at 2 h, while urolithin metabolites peaked at 24 h, which may explain these vascular effects. Similarly, 50 g of freeze-dried strawberry powder improved serum nitrate/nitrite concentrations, which positively influenced reactive hyperemia, a measure of blood flow, in young obese males after one week [287]. These results reflected positive changes in reactive hyperemia in pre-hypertensive postmenopausal females, in which 25 g/d of freeze-dried strawberry was efficacious after eight weeks [270]. In mixed-sex studies, black raspberry appears particularly efficacious in improving vascular function in both hypertensive subjects and subjects with metabolic syndrome [147,288,289]. Studies investigating the effect of berry polyphenols on vascular function variables in premenopausal females alone or systematically comparing outcomes of female and male participants are scant; however, there is strong evidence to suggest berries and their polyphenols have beneficial effects in reducing oxidative stress and improving vascular function in humans.

Although sex differences in vascular function in response to berry polyphenols have not been fully elucidated, it is likely that sex differences exist. For example, red wine polyphenols (which include gallic acid, caffeic acid, and anthocyanins) induced endothelium-dependent vasodilation in aortas from female and male rats, but relaxation was more pronounced in female aortic rings than those from males [290]. This effect was shown to be mediated exclusively by NO, via inclusion of a NOS inhibitor [290]. The addition of non-specific ER-antagonist ICI in this study did not affect red wine polyphenol-induced aortic relaxation in either sex, but it reduced E2-induced aortic relaxation [290]. It is important to note that the effect of ICI on E2-induced relaxation was only tested in female aortas in this study, and that the non-specific antagonistic nature of ICI does not allow for the assessment of the isolated impact of ERα or ERβ. This suggests red wine polyphenols impact endothelium-dependent vasodilation and endothelial NO production in an ER-independent manner (Figure 2). In this investigation, red wine polyphenols appear to elicit this effect via mediation of antioxidant enzymes, including SOD and catalase (evidenced by addition of SOD and catalase analogues) and by increasing phosphorylation of eNOS through the PI3K/Akt pathway [290]. Therefore, some sex-specific effects of polyphenols may be due to mediation of ROS. Considering decreased FMD in females at risk for CVD compared to male counterparts [291], these sex-specific effects of polyphenols in vascular function warrant further investigation in humans.

#### 4.4.3. Potential ROS-Independent Effects of Berry Polyphenols

While ROS can independently mediate inflammatory signaling (MAPKs and NF-κB), such as by modifying redox sensitive IKK and MEK [292], polyphenols likely have a multi-targeted role, inhibiting ROS and inflammation independent of each other. For example, in a pressure-overload model of HF in male mice, myricetin (200 mg/kg/d for six weeks) administration resulted in significantly reduced MAPK and NF-κB phosphorylation and increased Nrf2 and HO-1 in cardiac tissue compared to sham animals [157]. However, Nrf2 silencing still resulted in reduced NF-κB and MAPK phosphorylation, which corresponded with comparable inhibition of cardiac hypertrophy with and without Nrf2 silencing. In vitro findings suggest that this anti-inflammatory effect occurred despite significant cellular oxidative stress in neonatal rat cardiomyocytes co-treated with H_2_O_2_, myricetin, and Nrf2 siRNA as indicated by increased 4-hydroxynonenal expression, a metabolite of lipid peroxidation, compared to control. It is likely that myricetin prevented MAPK and NF-κB activation at multiple upstream sites, namely reduced TRAF6 degradation, preventing TAK1 activation. Nonetheless, these results do not preclude oxidative modification of IKK and MEK, suggesting that this polyphenol may also inhibit these enzymes independent of ROS. Further experimental details for some of the investigations cited in this section are displayed in Supplementary Table S3.

## 5. Cardiac and Vascular Remodeling

Cardiac and vessel architecture and plasticity largely reflect function; thus, pathological remodeling of the vasculature can result in CVDs, including HF, hypertension, and atherosclerosis [9,293,294]. Apoptotic cells must be replaced to maintain cardiac and vessel structure. In the heart, remodeling of the myocardium begins with apoptosis, leading to subsequent fibrosis, and causing impaired LV function. Similarly, in arteries, hypertrophic remodeling is characterized by VSMC apoptosis and fibrosis, impairing vessel dilation and promoting cardiac ischemia. Thus, cardiac and vascular remodeling induced by apoptosis and fibrosis are key features of CVDs and represent a therapeutic target.

### 5.1. Apoptosis

Apoptosis involves either the extrinsic pathway due to cytokine signaling (e.g., TNFR1) or the intrinsic pathway due to intracellular damage, such as DNA or mitochondrial damage [295]. Under pathological conditions observed in CVD, it is likely that both are of relevance and significant overlap and convergence exists between pathways. In the inner mitochondrial membrane, cytochrome c (Cyt c) is a phospholipid-bound heme protein that facilitates electron transfer between complex III and IV of the electron transport chain. Under conditions of oxidative stress, mitochondrial membrane permeability increases, allowing release of Cyt c into the cytosol [296]. Upon cytosolic translocation, Cyt c in conjunction with ATP (to a greater extent dATP) allosterically activate apoptosis-protease activating factor 1, facilitating caspase-9 activation due to proteolytic cleavage [297]. Subsequently, caspase-9 facilities caspase-3 activation via cleavage, leading to cell death [298]. Illustrating the role of caspase, caspase inhibition following myocardial infarct in rats reduced LV cardiomyocyte apoptosis, reduced fibrosis, and preserved LV function [299]. A regulator of these apoptotic effects is the cytosolic translocation and oligomerization of both Bax and Bcl-2 homologous antagonist killer (Bak) on the mitochondria, in which mitochondrial membrane transition pore opening occurs, facilitating Cyt c release [295]. The transcriptional regulation of Bax, and Bak to a lesser extent, is regulated by p53 [300,301], which can be activated by oxidative stress [302]. Indeed, patients with HF have significantly greater p53 protein expression in their hearts compared with healthy controls [303]. Following transcription and synthesis, Bax and Bak can be activated along the intrinsic pathway due to DNA damage and can also be activated extrinsically by caspase-8 or JNK due to inflammatory cytokine signaling via TNFR [295,304]. To counteract these effects, anti-apoptotic protein Bcl-2 can inhibit caspase-8 activity, and Bcl extra-large (Bcl-xL) can prevent the oligomerization of Bax and Bak. Thus, Bcl-2 and Bcl-xL antagonize Bax/Bak apoptotic effects.

There are marked sex differences in cell survival and cell death, where males show significantly more age-related [305] and HF-related [306] cardiomyocyte loss, post-infarct apoptosis [307], and I/R induced apoptosis [308,309,310] than females. One mechanism that contributes to differences in cell survival between males and females is the PI3K/Akt pathway. Akt activation inhibits cardiomyocyte apoptosis [311] and reduces I/R injury [312] in in vitro and in vivo models of HF. Akt interferes with pro-apoptotic pathways by inhibiting p38MAPK (a pro-apoptotic inducer [304]), p53, and caspases [313]. Importantly, premenopausal females have elevated levels of phosphorylated Akt localized in cardiomyocyte nuclei compared to age-matched males and postmenopausal females [314]. Akt phosphorylates murine double minute 2 (MDM2), causing MDM2 nuclear translocation and consequent p53 ubiquitination and degradation [315,316,317,318]. MDM2 and p53 form a negative-feedback loop, as p53 increases the expression of MDM2, which leads to p53 degradation [319]. Akt also phosphorylates caspase-9, which inhibits caspase-9 cleavage [320]. E2 is an activator of the PI3K/Akt pathway [314,321] and induces nuclear translocation of Akt in cardiomyocytes [319]. Testosterone has also been indicated to increase Akt phosphorylation [322], but some models show refuting results, where acute testosterone infusion has exhibited a downregulation of Akt [323] and exacerbated cardiac damage [324,325] in I/R models. E2 also modulates p38 isoforms [326] and inhibits p53 activity [327]. In ovariectomized female rats, there is increased activated caspase-8, -9, and -3, and cytosolic Cyt c compared to female controls [328], which is reversed by E2 treatment [329,330]. Further, ovariectomized female SHR showed increased activated caspase-9 and activated caspase-3 compared to wild-type and sham-operated female SHRs [331]. Pro- and anti-apoptotic protein content also appears to differ by sex. For instance, under physiologic conditions, male rats show greater expression of anti-apoptotic Bcl-2 protein than female rats, but Bax and phosphorylated p38MAPK content was similar between sexes [332]. Additionally, following I/R, Bcl-2 protein decreased in male rats, Bax decreased in female rats, and phosphorylated p38 increased in male rats [332].

### 5.2. Fibrosis

In an inflammatory state, the endothelium and heart release chemoattractant molecules, which guide leukocytes, such as monocytes and neutrophils, to infiltrate these tissues [333,334]. Monocytes differentiate into macrophages to ingest apoptotic and necrotic cells and release the cytokine transforming growth factor (TGF)-β [335]. Cardiomyocytes and VSMCs also harbor the inactive form of TGF-β, which can be activated by ROS [336,337,338]. TGF-β is a key initiator of the fibrotic process by acting on fibroblasts that reside interstitially between cardiomyocytes, while arterial fibroblasts migrate inwards from the tunica adventitia [339]. TGF-β causes fibroblast differentiation into myofibroblasts, which have muscle cell phenotype characterized by α-smooth muscle actin expression [340]. In the heart, myofibroblasts disrupt the delicate architecture of the extracellular matrix between cardiomyocytes due to excessive collagen synthesis and deposition of matrix metalloproteinases (MMPs), disrupting coordinated contraction between cells and severely diminishing the functional capacity of the heart and hampering vessel plasticity [339,341].

ERs and ARs sex-specifically regulate fibrosis, collagen and MMP synthesis [342]. Sex differences in cardiac fibrosis have been reported in a number of cardiovascular conditions, including hypertension and atherosclerosis [343,344,345]. Male cardiac fibroblasts portray greater activation of collagen synthesis, greater collagen deposition, and result in a higher degree of fibrosis than that of females [342]. In response to E2 treatment, female rats showed downregulation of cardiac fibroblast collagen I and III expression, but both were upregulated in male rats [345,346]. This was similarly shown in human cardiac fibroblasts [345]. Mediation appears to be ER subtype specific as well, where an ERα-agonist is suppressive and an ERβ-agonist is stimulating in female versus male rat cardiac fibroblasts, respectively [345]. E2 also phosphorylates ER subtypes differentially in rat cardiac fibroblasts, where it phosphorylates ERα in females and ERβ in males [345]. Further, E2 inhibits MMP-2 via an ERα-mediated reduction in gene transcription in rat cardiac and human fibroblasts [347].

### 5.3. Berry Polyphenols in Cardiac and Vascular Remodeling

Berries are likely efficacious in the reduction of CVD-associated pathologic remodeling. For example, in a 2% blueberry diet in male Fischer-344 rats, ischemic HF was induced by coronary artery ligation [348]. The necrotic area of the myocardial infarction was significantly reduced in blueberry-supplemented animals compared to control. Further, blueberry supplementation significantly decreased apoptotic cells in myocardial sections, with 2% apoptotic cardiomyocytes detected compared to 9% detected in control. Berry polyphenols likely exert protective effects at nearly every stage of remodeling and act on a number of cellular pathways involved in these processes. In H9c2 cardiomyocytes, 24 h treatment of 10 ng/mL of anthocyanidins, including cyanidin-3-galactoside, cyanidin-3-glucoside, and cyanidin-3-arabinoside, preceded 600 μM/L H_2_O_2_ for 2 h [349]. Single anthocyanidins decreased H_2_O_2_-induced apoptosis, although their combination was more effective. Berry polyphenols likely target p53, upstream from Bax, to reduce apoptosis. For example, in rat VSMCs (sex unspecified), 200 μg/mL of blackberry, raspberry, and black raspberry polyphenolic extract for 24 h followed by 100 nM of Ang II for 72 h resulted in a significant reduction in p53 protein expression, O_2_^−^ and H_2_O_2_ production, and p38MAPK and ERK1/2 phosphorylation [350]. As such, we would expect decreased Bax, and indeed, HUVECs pretreated with 50 μmol/L of quercetin for 30 min followed by 250 μmol/L H_2_O_2_ displayed significant decreases in DNA fragmentation, Bax, and cleaved caspase-3 compared to treatment with H_2_O_2_ alone [351]. Further, H_2_O_2_ decreased Bcl-2 and was restored with quercetin. In neonatal rat cardiomyocytes (donor sex unspecified), 3 h of anoxia (95% N_2_ and 5% CO_2_) significantly increased caspase-3 activity and apoptosis but was markedly reduced by resveratrol in a dose-dependent manner [154]. Thus, in multiple cells of the cardiovascular system, apoptotic signaling is reduced with berry polyphenols. These anti-apoptotic effects in vitro are reproducible in vivo. For example, in gallic acid-treated male SHR (1% of drinking water), cardiac Bax and cleaved caspase-3 expression was significantly reduced compared to control animals [352]. Additionally, in urolithin B pretreated H9c2 cells that underwent 3 h of hypoxia and 3 h of reoxygenation, urolithin B significantly reduced cleaved caspase-3 and apoptosis; however, Nrf2 silencing neutralized these effects [278]. This suggests a clear role for ROS in mediating apoptosis [278], but polyphenols may also act in ROS-independent manners [157].

In addition to apoptotic signaling, berry polyphenols also mediate fibrotic processes. For example, in male mice that underwent trans-aortic constriction to induce HF (pressure-overload model), 100 mg/kg/day of gallic acid or medications used in the treatment of HF, including 3 mg/kg/day of losartan (AT_1_R blocker), 1 mg/kg/day carvedilol (β-blocker), or 3 mg/kg/day furosemide (3 mg/kg/day), for two weeks following confirmed HF resulted in a reduction of cardiac collagen I and α-smooth muscle actin to a large extent with gallic acid treatment but minimally with medications [353]. In accordance with these findings, cardiac perivascular fibrosis was dramatically reduced with gallic acid treatment but not with medications. Rutin and quercetin appear to have TGF-β reducing effects in an isoproterenol model of HF in male Wistar rats [354]; however, polyphenols likely directly prevent differentiation of fibroblasts to myofibroblasts even with pathological stimulus. For example, resveratrol pretreatment (25 μM) for 30 min significantly reduced α-smooth muscle actin in Ang II- (100 nM) and TGF-β-treated (200 pM) cardiac fibroblasts derived from male rats [355]. Interestingly, despite 3 h pretreatment with 5 ng/mL TGF-β, gallic acid was still able to halt differentiation of fibroblasts as evidenced by decreased α-smooth muscle actin and decreased collagen I protein expression [353]. While human studies that assess berries or berry polyphenols in pathological vascular and cardiac remodeling are lacking, the consumption of cranberry juice for twelve weeks (increasing in quantity from 125 mL at baseline to 500 mL at twelve weeks) resulted in a significant reduction of serum MMP-9 concentrations in normotensive and pre-hypertensive males [356]. Based on promising preclinical findings, it is likely that berries and their polyphenols can mitigate the pathological processes of vascular and cardiac remodeling. Further experimental details for some of the investigations cited in this section are displayed in Supplementary Table S4.

## 6. Conclusions

Based on promising preclinical studies and limited human data, berry polyphenols likely target many of the underlying cellular drivers of CVD, including inflammation, oxidative stress, and pathological remodeling. Systematic investigation of sex differences on the effects of berry polyphenols on cardiovascular health is currently limited. Additional studies are needed to elucidate mechanistic differences in the effects of polyphenols on the pathological pathways of CVD between and within sexes. Despite the paucity of data explicitly investigating sex differences, there is a substantial amount of research regarding two factors that support the hypothesis that polyphenols exert sex-specific effects, including (1) the effects of estrogen and estrogen-signaling in the human cardiovascular system and (2) the effects of berry-derived polyphenols and their cellular targets, which appear to mimic that of endogenous estrogen. Considering the increased risk of CVD in males versus females, increased berry consumption in males should be encouraged for cardio-protection. However, the complex nature of androgens and cardiovascular health remains incompletely understood, yielding this as an unresolved question. Continued research into the sex-specific effects of sex hormones, ERs, and ARs in cardiovascular health is warranted alone and in conjunction with polyphenol interventions. Regardless of a sex-specific effect, berry consumption, due to their rich polyphenolic profile, represents a promising interventional tool in the treatment and prevention of CVD.

## Supplementary Materials

The following are available online at https://www.mdpi.com/2072-6643/13/2/387/s1: Table S1: Search terms used for study compilation, Table S2: Berry Polyphenols and Inflammation, Table S3: Berry Polyphenols and Oxidative Stress, Table S4: Berry Polyphenols and Pathological Remodeling.
