# Supplementary material for: Berry-Derived Polyphenols in Cardiovascular Pathologies: Mechanisms of Disease and the Role of Diet and Sex
Source: Nutrients. 2021 Jan 27;13(2):387. doi: 10.3390/nu13020387 (PMC7911141; doi:10.3390/nu13020387)
Supplement: Supplementary file 1 [file nutrients-13-00387-s001.pdf]

nutrients

MDPI

Supplementary

Table S1. Search terms used for study compilation.

Title/Abstract 1st term	AND	Title/Abstract 2nd term	AND	Title/Abstract 3rd term
Strawberry		Cardiovascular disease		Sex differences
Blackberry		Coronary artery disease		Sex
Raspberry		Inflammation		Gender
Blueberry		Oxidative stress		
Berries		Vascular		
Polyphenol		Hypertension		
Flavonoid		Heart Failure		
Anthocyanin		Hypertrophy		
Flavanol		Fibrosis		
Flavonol		Endothelial		
Phenolic acid		Vascular smooth muscle cell		
Gallic acid		Cardiomyocyte		
Ellagic acid		MMP		
Caffeic acid		Nrf2		
Cyanidin		Sirt1		
Delphinidin		Nox		
Epicatechin		eNOS		
Resveratrol		TLR4		
Quercetin		TNFR		
Kaempferol		Apoptosis		
Myricetin		Bcl-2		
Urolithin		Bax		
Vanillic acid		p53		
		Estrogen		
		Metabolism		
		Microbiota		

Table S2. Berry Polyphenols and Inflammation.

Author	Subject Characteristics	Intervention	Duration	Design	Findings
Aghababaee, et al. 2015 [1]	54 males and 18 females with dyslipidemia	300 mL/day blackberry juice with pulp	8 weeks	Randomized trial	↓ *hs*-CRP
An JH, et al. 2016 [2]	13 males and 31 females with prediabetes	900 or 1800 mg per day of black raspberry extract	12 weeks	Randomized, double-blind, placebo-controlled study	↓ MCP-1
Basu A, et al. 2009 [3]	16 obese females with metabolic syndrome	25 g/d freeze-dried strawberry	4 weeks	Repeated measures	↔ *hs*-CRP
Basu A, et al. 2010a [4]	44 obese females and 4 obese males (~50 yo) with metabolic syndrome	50 g/d freeze-dried blueberry	8 weeks	Randomized, single-blind, placebo-controlled trial	↔ *hs*-CRP, IL-6, ICAM-1, or VCAM-1
Basu A, et al. 2010b [5]	25 females and 2 males with metabolic syndrome and obesity	50 g/d freeze-dried strawberry	8 weeks	Randomized study	↓ VCAM-1
Basu A. et al. 2014 [6]	5 males and 55 females with obesity	50 or 25 g/d freeze-dried strawberry	12 weeks	Randomized study	↔ *hs*-CRP, VCAM-1, ICAM-1
Jeong HS, et al. 2014 [7]	36 males and 41 females with metabolic syndrome	750 mg/d black raspberry	12 weeks	Randomized, placebo-controlled trial	↔ *hs*-CRP, ICAM-1, VCAM-1<br>↓ IL-6 and TNF-α
Jeong HS, et al. 2016 [8]	24 males and 21 females with pre-hypertension	1,500 or 2,500 mg/d black raspberry	8 weeks	Randomized, double-blinded, placebo-controlled clinical trial	↔ *hs*-CRP, ICAM-1, VCAM-1<br>↓ IL-6 and TNF-α
Jeong HS, et al. 2016 [9]	51 middle aged females and males with metabolic syndrome	750 mg/d freeze-dried black raspberry	12 weeks	Randomized, placebo-controlled trial	↓ IL-6 and TNF-α
Johnson SA, et al. 2015 [10]	48 postmenopausal females (45-65 years old) with pre- or stage 1-hypertension	22 g/d freeze-dried blueberry powder	8 weeks	Randomized, double-blind, placebo-controlled clinical trial	↔ *hs*-CRP
Karlsen A, et al. 2010 [11]	46 males (30-70 yo) and 17 postmenopausal females (45-70 yo) with at least one CVD risk factor	330 ml/d bilberry juice	4 weeks	Parallel group, randomized, placebo-controlled trial	↓ *hs*-CRP, ↓ IL-6, ↑ TNF-α

Lehtonen HM, et al. 2011 [12]	110 overweight and obese, premenopausal females	Whole, frozen bilberries (BB)<br>Air dried sea buckthorn (SB)<br>SB phenolic extract (SBe)<br>SB oil (SBo)<br>Content equal to 100 g fresh berries	33-35 d intervention followed by 30-39 d wash-out	Randomized, crossover design	↓ VCAM-1 after BB and SBo<br>↓ ICAM-1 after SBe<br>↓ TNF-*α* after BB, SB, and SBe
Nair AR, et al. 2017 [13]	9 males and 18 females (53-62 yo) with metabolic syndrome	45 g/d freeze-dried blueberry powder	6 weeks	Randomized, double-blind, placebo-controlled clinical trial	↓ mRNA of TNF-*α*, TLR4, and IL-6
Schell J, et al. 2019 [14]	5 males and 20 females with type II diabetes	250 g fresh raspberry	4 weeks	randomized crossover study	↔ *hs*-CRP, IL-1β<br>↓ IL-6, TNF-*α*
			Postprandial	randomized crossover study	↔ *hs*-CRP, IL-1β<br>↓ IL-6, TNF-*α*
Bhaskar S, et al. 2016 [15]	Female, Sprague-Dawley rats	25 mg/kg Quercetin	60 d	High-cholesterol diet	↓ *hs*-CRP, ↓ IL-6 mRNA
Liao HH, et al. 2019 [16]	8-10-week-old, male mice (C57BL/6)	200 mg/kg/d myricetin	6 weeks	Pressure overload-induced HF	↓ NF-κB and MAPK phosphorylation (with and without Nrf2 knockdown), ↓ TRAF6 expression and ubiquitination, ↓ TAK1 phosphorylation
Zhang C, et al. 2012 [17]	1-3-day-old Sprague-Dawley rats (gender unspecified)	5, 10, or 20 μM resveratrol	Applied<br>5 min after reoxygenation	A/R injury model (3 h anoxia, 2 h reoxygenation)	↓ TLR4 mRNA and protein, ↓ NF-κB p65, ↑ IκB*α*, ↓ TNF-*α*, ↓ IL-1β
Bhaskar S, et al. 2016 [15]	HUVEC	Quercetin	1 h	24 h 50 μM/ml oxidized LDL	↓ VCAM-1, ↓ ICAM-1, ↑ cytosolic NF-κB p65, ↓ nuclear NF-κB p65, ↓ TLR4 mRNA
Feresin RG, et al. 2016 [18]	Rat aortic VSMC (male donor)	200 μg/mL polyphenolic extract of freeze-dried blackberries (BL), raspberries (RB), and black raspberries (BRB)	24 h	72 h 100 nM Ang II	↓ Ang II-induced ERK1/2 and p38MAPK phosphorylation by all berry extracts<br>↓ Ang II-induced Akt phosphorylation by BL and BRB, but not RB

Huang WY, et al. 2014 [19]	HUVEC	1, 10, 50, or 100 μM Malvidin-3-glucoside (Mv-3-glc) Malvidin-3-galactoside (Mv-3-gal) Mv-3-glc + Mv-3-gal	18 h	6 h 10 μg/L TNF-α	Mv-3-glc generally showed a stronger inhibitory effect than Mv-3-gal 50 and 100 μM Mv-3-glc inhibited ICAM-1 and VCAM-1 by >50%, all concentrations ↓ MCP-1 and ICAM-1 mRNA 50 and 100 μM Mv-3-gal inhibited ICAM-1 and VCAM-1 by > 50%, all concentrations ↓ ICAM-1 mRNA, 50 and 100 μM ↓ MCP-1 mRNA More effective at inhibiting ICAM-1 in combination versus isolation, all concentrations ↓ MCP-1 and ICAM-1 mRNA All concentrations (in combination and isolation) ↓ IκBα degradation
Hung CH, et al. 2015 [20]	HUVEC	10 μM quercetin	1 h	150 μM/ml oxidized LDL	↓ NF-κB activity, ↓ IL-8 secretion, ↓ ICAM-1, ↓ VCAM-1
Karlsen A, et al. 2010 [11]	Human monocytic cell line	25 and 50 μmol/L quercetin 50 μmol/L resveratrol 25 μmol/L epicatechin 50 μmol/L myricetin 50 μmol/L petunidin 50 μmol/L delphinidin	30 min	6 h 1 μg/ml LPS	Quercetin, resveratrol, and epicatechin ↓ NF-κB myricetin, petunidin and delphinidin ↑ NF-κB
Li T, et al. 2019 [21]	Murine macrophage RAW 264.7 cells	6 μM Quercetin + 150 μM Catechin or	24 h	24 h 1 μg/ml LPS	Quercetin - ↓ TNF-α, ↓ IL-1β, ↓ p-p38MAPK, ↓ p-ERK, ↓ TLR4 mRNA, ↑

		3 μM Quercetin + 75 μM Catechin			MyD88 mRNA, ↓ TRAF6 mRNA, ↑ TAK1 mRNA<br><br>Catechin - ↓ TNF-α, ↓ IL-1β, ↓ p-JNK, ↓ p-p38MAPK, ↓ p-ERK, ↓ TLR4 mRNA, ↓ TRAF6 mRNA, ↑ TAK1 mRNA<br>Quercetin + Catechin – ↓↓ TNF-α, ↓↓ IL-1β, ↓ NF-κB p65, ↓ p-JNK, ↓ p-p38MAPK, ↓ p-ERK, ↓ TLR4 mRNA, ↓ MyD88 mRNA, ↓ TRAF6 mRNA, ↑ TAK1 mRNA
Nwachukwu JC, et al. 2014 [22]	MCF-7 breast cancer cells	10 μM Resveratrol	24 h	24 h 1 ng/ml TNF-α	Suppressed TNF-α-induced ↑ in IL-6 (similar to E2) – reversed by ICI, indicating ER-dependent mechanism<br>Almost all E2-regulated genes were modulated by resveratrol<br>Genes that were altered in the opposite direction as of TNF-α activity were more sensitive to resveratrol than E2
Olson ER, et al. 2005 [23]	Cardiac fibroblasts from male Sprague-Dawley rats	25 μM resveratrol	30 min	100 nM Ang II or 200 pM TGF-β	↓ phosphorylation of MEK and ERK1/2
Sivasinprasasn S, et al. 2016 [24]	HUVEC (EA.hy926)	5, 10, or 20 μM C3G	2 h	C3G exposure, followed by 24 h 1 μM Ang II exposure	↑ IκBα, ↓ total NF-κB p65, ↑ cytosolic NF-κB p65, ↓ nuclear NF-κB p65

↑ denotes an increase, ↓ denotes a decrease, and ↔ indicates no change. Abbreviations: E2, estradiol; ERL extracellular receptor kinase; hs-CRP, high-sensitivity C-reactive protein; ICAM-1, intracellular adhesion molecule 1; IκBα, inhibitor of nuclear factor kappa B; IL-6, interleukin-6; MCP-1, monocyte chemoattractant

protein-1; MAPK, mitogen-activated protein kinase; MEK, mitogen-activated protein kinase kinase; Myd88, myeloid differentiation primary response 88; NF-κB, nuclear factor kappa-light-chain-enhancer of activated B cells; TAK1, transforming growth factor beta-activated kinase 1; TLR4, toll-like receptor-4; TNF-α, tumor necrosis factor alpha; TRAF6, TNF receptor associated factor 6; VCAM-1, vascular cell adhesion molecule 1.

Table S3. Berry Polyphenols and Oxidative Stress.

Author	Subject Characteristics	Intervention	Duration	Design	Findings
Aghababaee, et al. 2015 [1]	54 males and 18 females with dyslipidemia	300 mL/day blackberry juice with pulp	8 weeks	Randomized trial	↓ SBP<br>↔ DBP
An JH, et al. 2016 [2]	13 males and 31 females with prediabetes	900 or 1800 mg per day of black raspberry extract	12 weeks	Randomized, double-blind, placebo-controlled study	↓ Oxidized LDL
Basu A, et al. 2009 [3]	16 obese females with metabolic syndrome	25 g/d freeze-dried strawberry	4 weeks	Repeated measures	↔ Oxidized LDL<br>↓ malondialdehyde and hydroxynonenal
Basu A, et al. 2010a [4]	44 obese females and 4 obese males (~50 yo) with metabolic syndrome	50 g/d freeze-dried blueberry powder	8 weeks	Randomized, single-blind, placebo-controlled trial	Greater ↓ in SBP, DBP, oxLDL, malondialdehyde, and hydroxynonenal than control group
Basu A, et al. 2014 [6]	5 males and 55 females with obesity	50 or 25 g/d freeze-dried strawberry	12 weeks	Randomized study	↔ SBP and DBP<br>↓ malondialdehyde and hydroxynonenal
Burton-Freeman B, et al. 2010 [25]	5 females and 10 males with hyperlipidemia	10 g freeze-died strawberry	12 weeks	Randomized single-blind, placebo-controlled crossover trial	↓ oxidized LDL
Djurica D, et al. 2016 [26]	25 obese or overweight males	50 g freeze-dried strawberry powder	Postprandial	Randomized, double-blind, cross-over	↑ NO levels
			1 week	Randomized, double-blind, cross-over	↔ SBP and DBP<br>↑ NO levels
Feresin RG, et al. 2017 [27]	71 pre- and stage 1-hypertensive postmenopausal females	50 or 25 g/d freeze-dried strawberry	4 and 8 weeks	Randomized double-blind, placebo controlled study	↓ arterial stiffness and SBP with 25 g/d<br>↑ NO levels with 50 g/d
Istas G, et al. 2018 [28]	10 healthy males	200 g and 400 g of red raspberries	2 h and 24 h	Postprandial	↑ FMD and arterial stiffness
Jeong HS, et al. 2016 [8]	24 males and 21 females with pre-hypertension	1,500 or 2,500 mg/d black raspberry	8 weeks	Randomized, double-blinded, placebo-controlled clinical trial	↔ DBP<br>↓ SBP

Jeong HS, et al. 2016 [9]	51 middle aged females and males with metabolic syndrome	750 mg/d freeze-dried black raspberry	12 weeks	Randomized, double-blinded, placebo-controlled clinical trial	↑ FMD
Johnson SA, et al. 2015 [10]	48 postmenopausal females (45-65 yo) with pre- or stage 1-hypertension	22 g/d freeze-dried blueberry powder	8 weeks	Randomized, double-blind, placebo-controlled clinical trial	↓ SBP and DBP, ↑ NO levels
Kay CD & Holub BJ. 2007 [29]	8 middle aged males	100 g freeze-dried wild blueberry powder	Repeated measures	Postprandial	↑ serum antioxidant capacity
Marniemi J, et al. 2000 [30]	40 sixty-year old males	100 g of bilberries, lingonberries, or black currants	8 weeks	Randomized	↑ serum antioxidant capacity
Mathison BD, et al. 2014 [31]	6 healthy males and 6 healthy females	16 oz. cranberry juice	Postprandial	Repeated measures	↑ SOD and GPx
Mazza G, et al. 2002 [32]	5 healthy males	100 g freeze-dried wild-blueberry powder	Postprandial	Repeated measures	↑ serum antioxidant capacity
McLeay Y, et al. 2012 [33]	10 healthy females	200 g frozen New Zealand blueberries	12 h and 36 h post exercise	Randomized cross-over study	↑ serum antioxidant capacity
Nair AR, et al. 2017 [13]	9 males and 18 females (53-62 yo) with metabolic syndrome	45 g/d freeze-dried blueberry powder	6 weeks	Randomized, double-blind, placebo-controlled clinical trial	↓ ROS, ↓ O_2^-
Netzel M, et al. 2002 [34]	3 healthy males and 3 healthy females	400 ml juice containing 30% white grape-, 25% blackcurrant-, 15% elderberry-, 10% sour cherry-, 10% blackberry- and 10% aronia-juice	Postprandial	Repeated measures	↑ serum antioxidant capacity<br>↓ malondialdehyde
Netzel M, et al. 2005 [35]	4 healthy males and 4 healthy females	200, 300 or 400 ml of elderberry juice	Postprandial	Repeated measures	↑ serum antioxidant capacity
Rodriguez-Mateos A, et al. 2013 [36]	21 healthy males (25-29 yo)	freeze-dried wild-blueberry powder<br>766, 1278, and 1791 mg total blueberry polyphenols	Time-course trial - assessment at baseline, and 1, 2, 4, and 6 h after consumption	Randomized, double-blind, placebo-controlled, crossover clinical trial	↑ postprandial FMD with all doses, peaked at 1-2 h and 6 h post-consumption<br>↓ Nox activity

		319, 637, 766, 1278, and 1791 mg total blueberry polyphenols	Dose-dependency trial – assessment at baseline, and 1 h after consumption	Randomized, double-blind, placebo-controlled, crossover clinical trial	Endothelial function ↑ linearly up to 766 mg, then plateaued/↓ slightly at higher intakes
Schell J, et al. 2019 [14]	5 males and 20 females with type II diabetes	250 g fresh raspberry	4 weeks	randomized crossover study	↔ small and large artery elasticity and DBP<br>↓ SBP
			Postprandial	randomized crossover study	↔ small and large artery elasticity, SBP and DBP
Tulipani S, et al. 2009 [37]	3 healthy males and 5 healthy females	1 kg of fresh strawberries	Postprandial	Repeated measures	↑ serum antioxidant capacity
Ding Y, et al. 2014 [38]	4-week-old, wild-type and ApoE$^{-/-}$, male mice (C57BL/6)	30 mg/kg/d ellagic acid	14 weeks	High-fat diet	↑ aortic Nrf2 and HO-1 expression, ↓ F2-isoprostane, ↑ NOS activity, ↑ endothelium-dependent vasodilation
Jin L, et al. 2017 [39]	4-week-old, male SHR	1% gallic acid in drinking water	16 weeks	Model of essential hypertension	↓ ACE and AT_1R expression, ↓ Nox activity and ↓ Nox1, Nox2, and Nox4 mRNA
Kalea AZ, et al. 2010 [40]	Weanling, male SHR	8% freeze-dried blueberry powder	7 weeks	Model of essential hypertension development	Promotes ↑ basal NO-dependent dilation and COX-mediated constriction<br>↑ endothelium-dependent dilation and vessel sensitivity
Kane M, et al. 2009 [41]	12-14-week-old, female and male Wistar rats	10^{-4} to 10^{-1} mg/ml red wine polyphenols (RWP) powder	Unspecified exposure time	Sex-specific assessment of vascular function	↑ RWP-induced endothelium-dependent relaxation due to ↑ antioxidant enzyme and ↑ phosphorylation of eNOS<br>↑ relaxation response in females vs. males

					ER-independent mechanism
Li M, et al. 2013 [42]	Male Wistar rats	25 or 50 mg/kg quercetin or rutin	21 d	Isoproterenol HF model (15 mg/kg)	Quercetin and rutin dose-dependently suppressed ↑ in malondialdehyde and ↓ in SOD activity
Liao HH, et al. 2019 [16]	8-10-week-old, male mice (C57BL/6)	200 mg/kg/d myricetin	6 weeks	Pressure overload-induced HF	↑ Nrf2, ↑ HO-1
Wang Y, et al. 2012 [43]	8-week-old, male $ApoE^{-/-}$ mice (C57BL/6)	2 g/kg cyanidin-3-glucoside (C3G)	8 weeks	High-fat, high-cholesterol diet	↓ aortic O_2^-, ↑ nitrite and nitrate, ↑ endothelium-dependent vasodilation, ↑ phosphorylated eNOS, ↑ cGMP
Yan X, et al. 2020 [44]	8-12-week-old, male mice (C57BL/6)	5 and 20 mg/kg/d gallic acid	2 weeks	Ang-II (490 ng/kg/min) induced hypertension	↓ BP, ↑ endothelium-dependent vasodilation, ↑ total and phosphorylated aortic eNOS
Zheng D, et al. 2020 [45]	7-week-old, male Sprague Dawley rats	0.7 mg/kg - intraperitoneal injection urolithin B	0, 24, and 48 h before surgery	I/R model of HF	↓ O_2^-, ↓ lipid peroxidation, restored SOD level, protected against p62 reductions, ↑ nuclear Nrf2, ↑ p62-Keap1 interaction, ↑ HO-1, ↑ NQO1
Hung CH, et al. 2015 [20]	HUVEC	2.5, 5, or 10 μM quercetin	24 h	150 μM/ml oxidized LDL	↑ Sirt1 expression, ↓ oxidized LDL-induced reductions of Sirt1 expression
		2.5, 5, or 10 μM quercetin<br>20 μM resveratrol	2 h	150 μM/ml oxidized LDL	Quercetin – ↑ Sirt1 expression in dose dependent manner<br>Resveratrol – greatest ↑ Sirt1 expression
		10 μM Quercetin	24 h	150 μM/ml oxidized LDL	↑ Nox2 and Nox4 expression, ↓ ROS (which was re-

					versed with SIRT1 silencing), protected against oxidized LDL-induced reductions of eNOS expression
Feresin RG, et al. 2016 [18]	Rat aortic VSMC	200 μg/mL polyphenolic extract of freeze-dried blackberries (BL), raspberries (RB), and black raspberries (BRB)	24 h	72 h 100 nM Ang II	↓ basal and Ang II-induced H_2O_2 by all berry extracts<br>↓ basal O_2^- by BL, but not RB or BRB<br>↓ Ang II-induced O_2^- by all berry extracts<br>↓ NADP/NADPH ratio by all berry extracts, but greatest ↓ by BL<br>↓ Nox1 expression by BL, but not RB or BRB<br>↔ Nox4 expression, regardless of berry extract<br>↔ Ang II-induced downregulation of catalase, regardless of berry extract<br>↑ basal and Ang II-treated SOD1 expression by all berry extracts<br>↑ basal and Ang II-treated SOD2 and GPx1 expression by RB and BRB, but not by BL
Sivasinprasasn S, et al. 2016 [24]	HUVEC (cell line EA.hy926)	5, 10, or 20 μM cyanidin-3-glucoside (C3G)	2 h	C3G exposure, followed by 24 h 1 μM Ang II exposure	↓ ROS, ↓ iNOS, ↑ total Nrf2 content, ↓ cytosolic Nrf2, ↑ nuclear Nrf2, ↑ HO-1, ↑ SOD
Xu JW, et al. 2007 [46]	Bovine artery and mouse kidney vascular endothelial cells	5 or 50 μM cyanidin	24 h	50 ng/mL TNF-α-induced endothelial cell apoptosis	↑ eNOS expression<br>Suppressed ↓ in intracellular glutathione

↑ denotes an increase, ↓ denotes a decrease, and ↔ indicates no change. Abbreviations: ACE, angiotensin converting enzyme; Ang II, angiotensin II; AT_1R, angiotensin II type-1 receptor; COX, cyclooxygenase; DBP, diastolic blood pressure; eNOS, endothelial nitric oxide synthase; FMD, flow-mediated dilation; GPx1, Glutathione peroxidase 1; H_2O_2, hydrogen peroxide; HO-1, heme oxygenase 1; iNOS, inducible nitric oxide synthase; KEAP1, kelch-like ECH-associated protein 1; NO, nitric oxide; Nox, NADPH-oxidase; Nrf2, nuclear factor erythroid 2-related factor 2; NQO1, NADPH quinone dehydrogenase 1; O_2^-, superoxide; ROS, reactive oxygen species; SBP, systolic blood pressure; Sirt1, sirtuins 1; SOD, superoxide dismutase.

Table S4. Berry Polyphenols and Pathological Remodeling.

Author	Subject Characteristics	Intervention	Duration	Design	Findings
Ruel G, et al. 2009 [47]	30 healthy, sedentary, abdominally obese males (41 – 61 yo)	Low-calorie cranberry juice cocktail<br>Bout 1 – 125 ml/d<br>Bout 2 – 250 ml/d<br>Bout 3 – 500 ml/d	12 weeks total (3 bouts of 4 weeks each)	Repeated measures trial	↓ plasma MMP9 (week 12 vs. baseline)<br>↓ in MMP9 was associated with ↑ plasma nitrate/nitrite
Ahmet I, et al. 2009 [48]	2-month-old, male Fischer-344 rats	2% blueberry-enriched diet	3 months prior to surgery and 10 weeks following surgery (half of each group crossed over to other diet)	Model of ischemic HF (coronary artery ligation)	↓ necrotic infarct area, ↓ apoptotic cell death
Jin L, et al. 2017 [39]	4-week-old, male SHR	1% gallic acid in drinking water	16 weeks	Model of essential hypertension	↓ aortic wall thickness compared to SHR with no gallic acid
Jin L, et al. 2018a [49]	4-week-old, male Wistar-Kyoto and SHR	1% gallic acid in drinking water	16 weeks	Model of essential hypertension	↓ cardiac hypertrophy,<br>↓ all CaMKII isoforms,<br>↓ caspase 3 expression,<br>↓ Bax expression and mRNA
Jin L, et al. 2018b [50]	6-week-old, male mice (CD-1)	100 mg/kg/d gallic acid (GA) compared to<br>losartan 3 mg/kg/d (L),<br>carvedilol 1 mg/kg/d (C),<br>or furosemide 3 mg/kg/d (F)	2 weeks following surgery	Aortic constriction HF model	GA ↓ perivascular fibrosis,<br>↓ collagen I expression,<br>↓ fibronectin, and<br>↓ MMP2 mRNA,<br>but ↔ with L, C, or F<br>↔ in MMP9 or MMP13 mRNA with GA, L, C, or F<br>GA ↓ TGF-β1 signaling
Yan X, et al. 2020 [44]	8-12-week-old, male mice (C57BL/6)	5 and 20 mg/kg/d gallic acid	2 weeks	Ang II (490 ng/kg/min) induced hypertension	↓ Ang II-induced aortic wall thickening and collagen deposition, ↓ collagen I and collagen III mRNA expression
Zhang C, et al. 2012 [17]	1-3-d-old Sprague-Dawley rats	5, 10, or 20 μM resveratrol	Applied 5 min after reoxygenation	A/R injury model (3 h anoxia, 2 h reoxygenation)	Dose-dependent ↓ in apoptotic cell death,

					↓ caspase 3 activity
Zheng D, et al. 2020 [45]	7-week-old, male Sprague Dawley rats	0.7 mg/kg - intraperitoneal injection urolithin B	0, 24, and 48 h before surgery	I/R model of HF	↓ apoptotic cell death, ↓ cleaved caspase 3
Choi YJ, et al. 2003 [51]	HUVEC	50 µmol/L quercetin<br>50 µmol/L catechin<br>50 µmol/L epigallocatechin gallate (EGCG)	30 min	30 min 250 µmol/L H_2O_2	Quercetin and EGCG ↓ H_2O_2-induced apoptotic cell death, but catechin did not<br>↓ DNA fragmentation by all treatments<br>Quercetin and EGCG restored Bcl-2, ↓ Bax, and ↓ cleaved caspase 3
Feresin RG, et al. 2016 [18]	Rat aortic VSMC	200 µg/mL polyphenolic extract of freeze-dried blackberries (BL), raspberries (RB), and black raspberries (BRB)	24 h	72 h 100 nM Ang II	↓ p53 protein expression by all berry extracts, but greatest ↓ by BL
Hung CH, et al. 2015 [20]	HUVEC	10 µM quercetin	24 h	150 µM/ml oxidized LDL	↑ Akt phosphorylation, ↓ cytosolic Cyt c
Isaak CK, et al. 2017 [52]	H9c2 rat cardiomyoblasts	10 ng/mL lingonberry anthocyanins (cyanidin-3-galactoside, cyanidin-3-glucoside, and cyanidin-3-arabinoside)	24 h	2 h 600 µM/L H_2O_2	↓ H_2O_2-induced apoptosis and ↓ caspase 3 activation in combination and isolation
Olson ER, et al. 2005 [23]	Cardiac fibroblasts from male Sprague-Dawley rats	25 µM resveratrol	30 min	100 nM Ang II or 200 pM TGF-β	↓ α-smooth muscle actin
Xu JW, et al. 2007 [46]	Bovine artery endothelial cells	5 or 50 µM cyanidin	24 h	50 ng/mL TNF-α-induced endothelial cell apoptosis	Dose-dependent ↓ TNF-α-induced cell death<br>Suppressed ↑ in cleaved caspase 3<br>↑ ERK1/2 and Akt phosphorylation

↑ denotes an increase, ↓ denotes a decrease, and ↔ indicates no change. Abbreviations: Akt, protein kinase B; Ang, angiotensin; Bax, Bcl-2-associated X protein; Bcl-2, B-cell lymphoma 2; Cyt C, cytochrome C; ERK1/2, extracellular receptor kinase; H_2O_2, hydrogen peroxide; MMP, matrix metalloproteinase; SHR, spontaneously hypertensive rats; TGF-β, transforming growth factor beta; TNF-α, tumor necrosis factor alpha.

References

1. Aghababaee, S.; Vafa, M.; Shidfar, F.; Tahavorgar, A.; Gohari, M.; Katebi, D.; Mohammadi, V. Effects of blackberry (Morus nigra L.) consumption on serum concentration of lipoproteins, apo A-I, apo B, and high-sensitivity-C-reactive protein and blood pressure in dyslipidemic patients. *J Res Med Sci* **2015**, *20*, 684-691, doi:10.4103/1735-1995.166227.
2. An, J.H.; Kim, D.L.; Lee, T.B.; Kim, K.J.; Kim, S.H.; Kim, N.H.; Kim, H.Y.; Choi, D.S.; Kim, S.G. Effect of Rubus Occidentalis Extract on Metabolic Parameters in Subjects with Prediabetes: A Proof-of-concept, Randomized, Double-blind, Placebo-controlled Clinical Trial. *Phytother Res* **2016**, *30*, 1634-1640, doi:10.1002/ptr.5664.
3. Basu, A.; Wilkinson, M.; Penugonda, K.; Simmons, B.; Betts, N.M.; Lyons, T.J. Freeze-dried strawberry powder improves lipid profile and lipid peroxidation in women with metabolic syndrome: baseline and post intervention effects. *Nutr J* **2009**, *8*, doi:10.1186/1475-2891-8-43.
4. Basu, A.; Du, M.; Leyva, M.J.; Sanchez, K.; Betts, N.M.; Wu, M.; Aston, C.E.; Lyons, T.J. Blueberries decrease cardiovascular risk factors in obese men and women with metabolic syndrome. *J Nutr* **2010**, *140*, 1582-1587, doi:10.3945/jn.110.124701.
5. Basu, A.; Fu, D.X.; Wilkinson, M.; Simmons, B.; Wu, M.; Betts, N.M.; Du, M.; Lyons, T.J. Strawberries decrease atherosclerotic markers in subjects with metabolic syndrome. *Nutr Res* **2010**, *30*, 462-469, doi:10.1016/j.nutres.2010.06.016.
6. Basu, A.; Betts, N.M.; Nguyen, A.; Newman, E.D.; Fu, D.; Lyons, T.J. Freeze-dried strawberries lower serum cholesterol and lipid peroxidation in adults with abdominal adiposity and elevated serum lipids. *J Nutr* **2014**, *144*, 830-837, doi:10.3945/jn.113.188169.
7. Jeong, H.S.; Hong, S.J.; Lee, T.B.; Kwon, J.W.; Jeong, J.T.; Joo, H.J.; Park, J.H.; Ahn, C.M.; Yu, C.W.; Lim, D.S. Effects of black raspberry on lipid profiles and vascular endothelial function in patients with metabolic syndrome. *Phytother Res* **2014**, *28*, 1492-1498, doi:10.1002/ptr.5154.
8. Jeong, H.S.; Hong, S.J.; Cho, J.Y.; Lee, T.B.; Kwon, J.W.; Joo, H.J.; Park, J.H.; Yu, C.W.; Lim, D.S. Effects of Rubus occidentalis extract on blood pressure in patients with prehypertension: Randomized, double-blinded, placebo-controlled clinical trial. *Nutrition* **2016**, *32*, 461-467, doi:10.1016/j.nut.2015.10.014.
9. Jeong, H.S.; Kim, S.; Hong, S.J.; Choi, S.C.; Choi, J.H.; Kim, J.H.; Park, C.Y.; Cho, J.Y.; Lee, T.B.; Kwon, J.W., et al. Black Raspberry Extract Increased Circulating Endothelial Progenitor Cells and Improved Arterial Stiffness in Patients with Metabolic Syndrome: A Randomized Controlled Trial. *J Med Food* **2016**, *19*, 346-352, doi:10.1089/jmf.2015.3563.
10. Johnson, S.A.; Figueroa, A.; Navaei, N.; Wong, A.; Kalfon, R.; Ormsbee, L.T.; Feresin, R.G.; Elam, M.L.; Hooshmand, S.; Payton, M.E., et al. Daily blueberry consumption improves blood pressure and arterial stiffness in postmenopausal women with pre- and stage 1-hypertension: a randomized, double-blind, placebo-controlled clinical trial. *J Acad Nutr Diet* **2015**, *115*, 369-377, doi:10.1016/j.jand.2014.11.001.
11. Karlsen, A.; Paur, I.; Bohn, S.K.; Sakhi, A.K.; Borge, G.I.; Serafini, M.; Erlund, I.; Laake, P.; Tonstad, S.; Blomhoff, R. Bilberry juice modulates plasma concentration of NF-kappaB related inflammatory markers in subjects at increased risk of CVD. *Eur J Nutr* **2010**, *49*, 345-355, doi:10.1007/s00394-010-0092-0.
12. Lehtonen, H.M.; Suomela, J.P.; Tahvonen, R.; Yang, B.; Venojarvi, M.; Viikari, J.; Kallio, H. Different berries and berry fractions have various but slightly positive effects on the associated variables of metabolic diseases on overweight and obese women. *Eur J Clin Nutr* **2011**, *65*, 394-401, doi:10.1038/ejcn.2010.26.
13. Nair, A.R.; Mariappan, N.; Stull, A.J.; Francis, J. Blueberry supplementation attenuates oxidative stress within monocytes and modulates immune cell levels in adults with metabolic syndrome: a randomized, double-blind, placebo-controlled trial. *Food Funct* **2017**, *8*, 4118-4128, doi:10.1039/c7fo00815e.
14. Schell, J.; Betts, N.M.; Lyons, T.J.; Basu, A. Raspberries Improve Postprandial Glucose and Acute and Chronic Inflammation in Adults with Type 2 Diabetes. *Ann Nutr Metab* **2019**, *74*, 165-174, doi:10.1159/000497226.
15. Bhaskar, S.; Sudhakaran, P.R.; Helen, A. Quercetin attenuates atherosclerotic inflammation and adhesion molecule expression by modulating TLR-NF-kappaB signaling pathway. *Cell Immunol* **2016**, *310*, 131-140, doi:10.1016/j.cellimm.2016.08.011.

16. Liao, H.H.; Zhang, N.; Meng, Y.Y.; Feng, H.; Yang, J.J.; Li, W.J.; Chen, S.; Wu, H.M.; Deng, W.; Tang, Q.Z. Myricetin Alleviates Pathological Cardiac Hypertrophy via TRAF6/TAK1/MAPK and Nrf2 Signaling Pathway. *Oxid Med Cell Longev* **2019**, *2019*, doi:10.1155/2019/6304058.
17. Zhang, C.; Lin, G.; Wan, W.; Li, X.; Zeng, B.; Yang, B.; Huang, C. Resveratrol, a polyphenol phytoalexin, protects cardiomyocytes against anoxia/reoxygenation injury via the TLR4/NF-kappaB signaling pathway. *Int J Mol Med* **2012**, *29*, 557-563, doi:10.3892/ijmm.2012.885.
18. Feresin, R.G.; Huang, J.; Klarich, D.S.; Zhao, Y.; Pourafshar, S.; Arjmandi, B.H.; Salazar, G. Blackberry, raspberry and black raspberry polyphenol extracts attenuate angiotensin II-induced senescence in vascular smooth muscle cells. *Food Funct* **2016**, *7*, 4175-4187, doi:10.1039/c6fo00743k.
19. Huang, W.Y.; Liu, Y.M.; Wang, J.; Wang, X.N.; Li, C.Y. Anti-inflammatory effect of the blueberry anthocyanins malvidin-3-glucoside and malvidin-3-galactoside in endothelial cells. *Molecules* **2014**, *19*, 12827-12841, doi:10.3390/molecules190812827.
20. Hung, C.H.; Chan, S.H.; Chu, P.M.; Tsai, K.L. Quercetin is a potent anti-atherosclerotic compound by activation of SIRT1 signaling under oxLDL stimulation. *Mol Nutr Food Res* **2015**, *59*, 1905-1917, doi:10.1002/mnfr.201500144.
21. Li, T.; Li, F.; Liu, X.; Liu, J.; Li, D. Synergistic anti-inflammatory effects of quercetin and catechin via inhibiting activation of TLR4-MyD88-mediated NF-kappaB and MAPK signaling pathways. *Phytother Res* **2019**, *33*, 756-767, doi:10.1002/ptr.6268.
22. Nwachukwu, J.; Srinivasan, S.; Bruno, N.; Parent, A.; Hughes, T.; Pollock, J.; Gjyshi, O.; Cavett, V.; Nowak, J.; Garcia-Ordonez, R., et al. Resveratrol modulates the inflammatory response via an estrogen receptor-signal integration network. *eLife* **2014**, *3*, doi:10.7554/eLife.02057.
23. Olson, E.R.; Naugle, J.E.; Zhang, X.; Bomser, J.A.; Meszaros, J.G. Inhibition of cardiac fibroblast proliferation and myofibroblast differentiation by resveratrol. *Am J Physiol Heart Circ Physiol* **2005**, *288*, H1131-1138, doi:10.1152/ajpheart.00763.2004.
24. Sivasinprasasn, S.; Pantan, R.; Thummayot, S.; Tocharus, J.; Suksamrarn, A.; Tocharus, C. Cyanidin-3-glucoside attenuates angiotensin II-induced oxidative stress and inflammation in vascular endothelial cells. *Chem Biol Interact* **2016**, *260*, 67-74, doi:10.1016/j.cbi.2016.10.022.
25. Burton-Freeman, B.; Linares, A.; Hyson, D.; Kappagoda, T. Strawberry modulates LDL oxidation and postprandial lipemia in response to high-fat meal in overweight hyperlipidemic men and women. *J Am Coll Nutr* **2010**, *29*, 46-54, doi:10.1080/07315724.2010.10719816.
26. Djurica, D.; Holt, R.; Ren, J.; Shindel, A.; Hackman, R.; Keen, C. Effects of a dietary strawberry powder on parameters of vascular health in adolescent males. *Br J Nutr* **2016**, *116*, 639-647, doi:10.1017/S0007114516002348.
27. Feresin, R.G.; Johnson, S.A.; Pourafshar, S.; Campbell, J.C.; Jaime, S.J.; Navaei, N.; Elam, M.L.; Akhavan, N.S.; Alvarez-Alvarado, S.; Tenenbaum, G., et al. Impact of daily strawberry consumption on blood pressure and arterial stiffness in pre- and stage 1-hypertensive postmenopausal women: a randomized controlled trial. *Food Funct* **2017**, *8*, 4139-4149, doi:10.1039/c7fo01183k.
28. Istas, G.; Feliciano, R.P.; Weber, T.; Garcia-Villalba, R.; Tomas-Barberan, F.; Heiss, C.; Rodriguez-Mateos, A. Plasma urolithin metabolites correlate with improvements in endothelial function after red raspberry consumption: A double-blind randomized controlled trial. *Arch Biochem Biophys* **2018**, *651*, 43-51, doi:10.1016/j.abb.2018.05.016.
29. Kay, C.; Holub, B. The effect of wild blueberry (Vaccinium angustifolium) consumption on postprandial serum antioxidant status in human subjects. *Br J Nutr* **2002**, *88*, 389-397, doi:10.1079/BJN2002665.
30. Marniemi, J.; Hakala, P.; Mäki, J.; Ahotupa, M. Partial resistance of low-density lipoprotein to oxidation in vivo after increased intake of berries. *Nutr Metab Cardiovasc Dis* **2000**, *10*, 331-337.
31. Mathison, B.; Kimble, L.; Kaspar, K.; Khoo, C.; Chew, B. Consumption of cranberry beverage improved endogenous antioxidant status and protected against bacteria adhesion in healthy humans: a randomized controlled trial. *Nutr Res* **2014**, *34*, 420-427, doi:10.1016/j.nutres.2014.03.006.

32. Mazza, G.; Kay, C.; Cottrell, T.; Holub, B. Absorption of anthocyanins from blueberries and serum antioxidant status in human subjects. *J Agric Food Chem* **2002**, *50*, 7731-7737, doi:10.1021/jf020690l.
33. McLeay, Y.; Barnes, M.; Mundel, T.; Hurst, S.; Hurst, R.; Stannard, S. Effect of New Zealand blueberry consumption on recovery from eccentric exercise-induced muscle damage. *J Int Soc Sports Nutr* **2012**, *9*, doi:10.1186/1550-2783-9-19.
34. Netzel, M.; Strass, G.; Kaul, C.; Bitsch, I.; Dietrich, H.; Bitsch, R. In vivo antioxidative capacity of a composite berry juice. *Food Res Int* **2002**, *35*, 213-216, doi:10.1016/S0963-9969(01)00186-7.
35. Netzel, M.; Strass, G.; Herbst, M.; Dietrich, H.; Bitsch, R.; Bitsch, I.; Frank, T. The excretion and biological antioxidant activity of elderberry antioxidants in healthy humans. *Food Res Int* **2005**, *38*, 905-910, doi:10.1016/j.foodres.2005.03.010.
36. Rodriguez-Mateos, A.; Rendeiro, C.; Bergillos-Meca, T.; Tabatabaee, S.; George, T.W.; Heiss, C.; Spencer, J.P. Intake and time dependence of blueberry flavonoid-induced improvements in vascular function: a randomized, controlled, double-blind, crossover intervention study with mechanistic insights into biological activity. *Am J Clin Nutr* **2013**, *98*, 1179-1191, doi:10.3945/ajcn.113.066639.
37. Tulipani, S.; Romandini, S.; Busco, F.; Bompadre, S.; Mezzetti, B.; Battino, M. Ascorbate, not urate, modulates the plasma antioxidant capacity after strawberry intake. *Food Chem* **2009**, *117*, 181-188, doi:10.1016/j.foodchem.2009.03.096
38. Ding, Y.; Zhang, B.; Zhou, K.; Chen, M.; Wang, M.; Jia, Y.; Song, Y.; Li, Y.; Wen, A. Dietary ellagic acid improves oxidant-induced endothelial dysfunction and atherosclerosis: role of Nrf2 activation. *Int J Cardiol* **2014**, *175*, 508-514, doi:10.1016/j.ijcard.2014.06.045.
39. Jin, L.; Piao, Z.H.; Sun, S.; Liu, B.; Kim, G.R.; Seok, Y.M.; Lin, M.Q.; Ryu, Y.; Choi, S.Y.; Kee, H.J., et al. Gallic Acid Reduces Blood Pressure and Attenuates Oxidative Stress and Cardiac Hypertrophy in Spontaneously Hypertensive Rats. *Sci Rep* **2017**, *7*, doi:10.1038/s41598-017-15925-1.
40. Kalea, A.Z.; Clark, K.; Schuschke, D.A.; Kristo, A.S.; Klimis-Zacas, D.J. Dietary enrichment with wild blueberries (Vaccinium angustifolium) affects the vascular reactivity in the aorta of young spontaneously hypertensive rats. *J Nutr Biochem* **2010**, *21*, 14-22, doi:10.1016/j.jnutbio.2008.09.005.
41. Kane, M.; Anselm, E.; Rattmann, Y.; Auger, C.; Schini-Kerth, V. Role of gender and estrogen receptors in the rat aorta endothelium-dependent relaxation to red wine polyphenols. *Vascul Pharmacol* **2009**, *51*, 140-146.
42. Li, M.; Jiang, Y.; Jing, W.; Sun, B.; Miao, C.; Ren, L. Quercetin provides greater cardioprotective effect than its glycoside derivative rutin on isoproterenol-induced cardiac fibrosis in the rat. *Can J Physiol Pharmacol* **2013**, *91*, 951-959, doi:10.1139/cjpp-2012-0432.
43. Wang, Y.; Zhang, Y.; Wang, X.; Liu, Y.; Xia, M. Supplementation with cyanidin-3-O-beta-glucoside protects against hypercholesterolemia-mediated endothelial dysfunction and attenuates atherosclerosis in apolipoprotein E-deficient mice. *J Nutr* **2012**, *142*, 1033-1037, doi:10.3945/jn.112.157701.
44. Yan, X.; Zhang, Q.Y.; Zhang, Y.L.; Han, X.; Guo, S.B.; Li, H.H. Gallic Acid Attenuates Angiotensin II-Induced Hypertension and Vascular Dysfunction by Inhibiting the Degradation of Endothelial Nitric Oxide Synthase. *Front Pharmacol* **2020**, *11*, doi:10.3389/fphar.2020.01121.
45. Zheng, D.; Liu, Z.; Zhou, Y.; Hou, N.; Yan, W.; Qin, Y.; Ye, Q.; Cheng, X.; Xiao, Q.; Bao, Y., et al. Urolithin B, a gut microbiota metabolite, protects against myocardial ischemia/reperfusion injury via p62/Keap1/Nrf2 signaling pathway. *Pharmacol Res* **2020**, *153*, doi:10.1016/j.phrs.2020.104655.
46. Xu, J.W.; Ikeda, K.; Yamori, Y. Inhibitory effect of polyphenol cyanidin on TNF-alpha-induced apoptosis through multiple signaling pathways in endothelial cells. *Atherosclerosis* **2007**, *193*, 299-308, doi:10.1016/j.atherosclerosis.2006.09.006.
47. Ruel, G.; Pomerleau, S.; Couture, P.; Lemieux, S.; Lamarche, B.; Couillard, C. Plasma matrix metalloproteinase (MMP)-9 levels are reduced following low-calorie cranberry juice supplementation in men. *J Am Coll Nutr* **2009**, *28*, 694-701, doi:10.1080/07315724.2009.10719803.

48. Ahmet, I.; Spangler, E.; Shukitt-Hale, B.; Juhaszova, M.; Sollott, S.J.; Joseph, J.A.; Ingram, D.K.; Talan, M. Blueberry-enriched diet protects rat heart from ischemic damage. *PLoS One* **2009**, *4*, doi:10.1371/journal.pone.0005954.
49. Jin, L.; Piao, Z.H.; Liu, C.P.; Sun, S.; Liu, B.; Kim, G.R.; Choi, S.Y.; Ryu, Y.; Kee, H.J.; Jeong, M.H. Gallic acid attenuates calcium calmodulin-dependent kinase II-induced apoptosis in spontaneously hypertensive rats. *J Cell Mol Med* **2018**, *22*, 1517-1526, doi:10.1111/jcmm.13419.
50. Jin, L.; Sun, S.; Ryu, Y.; Piao, Z.H.; Liu, B.; Choi, S.Y.; Kim, G.R.; Kim, H.S.; Kee, H.J.; Jeong, M.H. Gallic acid improves cardiac dysfunction and fibrosis in pressure overload-induced heart failure. *Sci Rep* **2018**, *8*, doi:10.1038/s41598-018-27599-4.
51. Choi, Y.J.; Kang, J.S.; Park, J.H.; Lee, Y.J.; Choi, J.S.; Kang, Y.H. Polyphenolic flavonoids differ in their antiapoptotic efficacy in hydrogen peroxide-treated human vascular endothelial cells. *J Nutr* **2003**, *133*, 985-991, doi:10.1093/jn/133.4.985.
52. Isaak, C.K.; Petkau, J.C.; Blewett, H.; O, K.; Siow, Y.L. Lingonberry anthocyanins protect cardiac cells from oxidative-stress-induced apoptosis. *Can J Physiol Pharmacol* **2017**, *95*, 904-910, doi:10.1139/cjpp-2016-0667.
